# Adam and the Ants: On the Influence of the Optimization Algorithm on the Detectability of DNN Watermarks

**DOI:** 10.3390/e22121379

**Published:** 2020-12-06

**Authors:** Betty Cortiñas-Lorenzo, Fernando Pérez-González

**Affiliations:** Atlanttic Research Center, University of Vigo, 36310 Vigo, Spain; bcortinas@gts.uvigo.es

**Keywords:** watermarking, deep neural networks, optimization algorithms, Adam, stochastic gradient descent, detectability

## Abstract

As training Deep Neural Networks (DNNs) becomes more expensive, the interest in protecting the ownership of the models with watermarking techniques increases. Uchida et al. proposed a digital watermarking algorithm that embeds the secret message into the model coefficients. However, despite its appeal, in this paper, we show that its efficacy can be compromised by the optimization algorithm being used. In particular, we found through a theoretical analysis that, as opposed to Stochastic Gradient Descent (SGD), the update direction given by Adam optimization strongly depends on the sign of a combination of columns of the projection matrix used for watermarking. Consequently, as observed in the empirical results, this makes the coefficients move in unison giving rise to heavily spiked weight distributions that can be easily detected by adversaries. As a way to solve this problem, we propose a new method called Block-Orthonormal Projections (BOP) that allows one to combine watermarking with Adam optimization with a minor impact on the detectability of the watermark and an increased robustness.

## 1. Introduction

Deep learning has substantially impacted technology over the last years, becoming an important center of attention for researchers all over the world. Such a great impact stems from the versatility it offers as well as the excellent results that DNNs achieve on multiple tasks, like image classification or speech recognition, which often reach and even surpass human-level performance [1,2].

However, far from being a simple task, the design of new DNNs is generally expensive not only in terms of human effort and time needed to build effective model architectures but mostly because of the large volume of suitable data that must be gathered and the vast amount of computational resources and power used for training. Consequently, businesses owning costly models are interested in protecting them from any illicit use, and this growing need has recently led researchers to a common concern on how to embed watermarks on DNNs. As a result, several frameworks for protecting the intellectual property of neural networks were proposed in the literature, and can be classified as black-box or white-box approaches. The first kind of methods (i.e., black-box) do not need to access model parameters for detecting the presence of watermarks. Instead, key inputs are introduced as special triggers to identify the original network, either by the use of the so-called adversarial examples [3] or backdoor poisoning [4].

White-box approaches, in contrast, directly affect the model parameters in order to embed watermarks. One of the most significant white-box contributions was proposed by Uchida et al. in [5,6], the algorithm under study in this paper. This watermarking framework employs a regularization term that defines a cost function for embedding the secret message. This regularizer, which transforms weights into bits by means of a projection matrix in a similar way to spread–spectrum techniques used in watermarking [7], is added to the main loss function and can be applied either at the beginning of the training phase (i.e., from scratch) or during fine-tuning steps.

On the other hand, when training a DNN, there are several optimization algorithms to choose from. With their pros and cons, some of the most popular are the classic Stochastic Gradient Descent (SGD) [8] and Adaptive Moment Estimation (Adam) [9]. We found that, in order to perform watermarking following the approach in [5,6], it is necessary to pay close attention to the optimization algorithm being used. In this paper, we prove that Adam, although being an appealing algorithm for lots of applications—especially because of its efficiency and training speed—poses a big problem when implementing this watermarking method.

As any other watermarking algorithm, it is important to meet some minimal requirements regarding fidelity, robustness, payload, efficiency, and undetectability [5,6,10]. This latter property involves the need for concealing any clue that would let unauthorized parties know if a watermark was embedded, along with its further consequences. In other words, from a steganographic point of view, watermarks should be embedded into the DNN without leaving any detectable footprint.

We show that, unlike SGD, Adam significantly alters the distribution of the coefficients at the embedding layer in a very specific way and, thus, it compromises the undetectability of the watermark. In particular, when using Adam, the histogram of the weights at the embedding layer shows that these coefficients tend to group together at both sides of zero, originating two visible spikes that grow in magnitude with the number of iterations, so that the initial distribution ends up changing completely. As we will see later, the update direction given by Adam depends on the projection matrix used for watermarking; specifically, it is based on the sign function, responsible for the symmetric two-spiked shape that emerges. This behavior is somewhat surprising, as with a pseudorandom projection matrix, one might expect that the weights evolve with random speeds; in contrast, the weights tend to move in unison in a way that is reminiscent of ants after they have established a solid path from the nest to a food source. Then, the statistical dependence of the weights with the projection matrix is weak, and this is mainly due to the information collapse induced by the sign function; this loss is invested in making the weights more conspicuous and, therefore, the watermark more easily detectable. As we will confirm, the sign phenomenon does not occur in other algorithms like SGD. The appearance of the sign was already pointed out and studied—without considering watermarking applications—by the authors in [11], who explained the adverse effects of Adam on generalization [12] as a consequence of this aspect. However, instead of delving into the general performance of the optimization algorithm itself, we show that the sign function which is involved in Adam’s update direction is detrimental for watermarking purposes, so we highlight the need for being careful with the selection of the optimization algorithm in these cases.

In this paper, we carry out a theoretical and experimental analysis and compare the results using Adam and SGD optimization. The analysis can be extended to other optimization algorithms. As a way to measure the similarity between the original weight distribution and the resulting one after the watermark embedding, we will use the Kullback–Leibler divergence (KLD) [13]. We will show that, as expected, when we use Adam, the KLD between both distributions is considerably larger than when we use SGD, thus confirming that, as opposed to SGD, Adam modifies the original distribution to a great extent. Furthermore, in order to perform this kind of watermarking and, at the same time, enjoy the advantages that Adam optimization provides, we propose a new method called Block-Orthonormal Projections (BOP), which uses a secret transformation matrix in order to reduce the detectability of the watermark generated by Adam. As we will see, BOP allows us to considerably reduce the KLD to small values which are comparable to those obtained with SGD. Therefore, we show that BOP allows us to preserve the original shape of the weight distribution.

In summary, this work makes the following two-fold contribution:

- We provide mathematical and experimental evidence for SGD and Adam to show that: (1) in contrast to SGD, the changes in the distribution of weights caused by Adam can be easily detected when embedding watermarks following the approach in [5,6] and, hence, (2) the use of Adam considerably increases the detectability of the watermark. For the purpose of carrying out this analysis, we use FFDNet [14]—a DNN that performs image denoising tasks—as the host network.
- We introduce a novel method based on orthogonal projections to solve the detectability problem that arises when watermarking a DNN which is being optimized with Adam. A side effect of this novel method is an increased robustness against weight pruning.

The remainder of this paper is organized as follows: Section 1.1 introduces the notation and Section 2 explains the frameworks and algorithms used in this study—host network, optimization algorithms, and watermarking method—in more detail. Section 3 presents the mathematical core that allows us to model the observed effects on the histograms of weights once the embedding process has finished. Then, in Section 4, we introduce BOP as a solution for using Adam and watermarking simultaneously, Section 5 presents the information-theoretic measures that we will implement, and Section 6 shows the experimental results. Finally, we point out some concluding remarks in Section 7. Two appendices give additional details on mathematical derivations (Appendix A) and the validity of certain assumptions (Appendix B).

### Notation

In this paper, we use the following notation. Matrices and vectors are denoted by upper-case and lower-case boldface characters, respectively, while random variables and their realizations are respectively represented by upper-case and lower-case characters.

For matrix and vector operations, we proceed as follows. As an example, let A be a matrix. Then, its transpose is denoted by $A^{T}$. Moreover, we use Tr[A] to represent the trace of A and ${\left(A\right)}_{i,j}$ to denote the (i,j)th element of A. $I_{N}$ refers to the N×N identity matrix. We use column vectors unless otherwise stated. In addition, we use 0 to denote a column vector of zeros and 1 for a column vector of ones. Let w be a column vector of length *N*; then, $\nabla_{w}$ is the gradient operator with respect to w that is:

$$\nabla_{w}≐{\left(\frac{\partial }{\partial w_{1}},\cdots ,\frac{\partial }{\partial w_{N}}\right)}^{T}$$

We use the operator ∘ to denote the Hadamard (i.e., sample-wise) product and ⊗ for the Kronecker product. Finally, E{·} and Var{·} denote the mathematical expectation and the variance, respectively.

## 2. Preliminaries

### 2.1. Host Network: FFDNet

The rapid development of Deep Learning over the last few years has led to new advances in the field of image restoration [15]. Several Convolutional Neural Networks (CNNs) have been designed to replace classical methods and, often, they offer new competitive advantages. This is the case of FFDNet [14], which performs image denoising tasks and is used in our work as the exemplary host network that is watermarked by means of the algorithm proposed in [5,6].

Image denoising is the task of removing noise from a given image. Let y be the input noisy image, x the clean image, and n the noise, which is usually modeled as zero-mean Additive White Gaussian Noise (AWGN), then we have y=x+n and we wish to obtain an estimate $\hat{x}$ of the clean image. As opposed to other CNNs for image denoising, FFDNet works on downsampled sub-images, and it is able to adapt to several noise levels using only a single network. For that purpose, a noise level map M is included also as an input, so that the function FFDNet aims to learn can be expressed as: $\hat{x}=F\left(y,M;w\right)$, where w represents the parameters of the network. Furthermore, FFDNet can handle spatially variant noise and offers competitive inference speed without sacrificing denoising performance [14].

**Figure 1:**
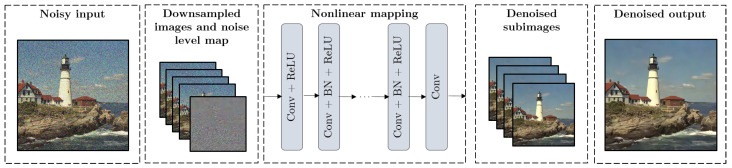
Architecture of the host network FFDNet.

Figure 1 shows the architecture of FFDNet. As we can see, it is composed of a downscaling operation on the input images, a nonlinear mapping consisting of Convolutional Layers, Batch Normalization steps [16], and ReLU activation functions; and, finally, an upscaling process to generate denoised images with the original size. Let $\left\{(y_{i},x_{i})\right\}$ be *L* noisy-clean image pairs from the training dataset, the denoising cost function is:

(1) $$f_{0}\left(w\right)=\frac{1}{2L}\sum_{i=1}^{L}||F\left(y_{i},M_{i};w\right)-x_{i}{\left|\right|}^{2}$$

FFDNet can be used for grayscale or color images. Table 1 shows the main differences between both configurations. As we can see, the total number of convolutional layers can be set to 15 or 12 for grayscale or RGB denoising, respectively. The number of feature maps and the size of the receptive field also differ, but this is not the case of the kernel size, which is kept to 3×3 for either grayscale or RGB. In this paper, we implement the RGB version of FFDNet and proceed as follows: (1) train FFDNet from scratch using Adam optimization without embedding any watermark and (2) fine-tune the network to embed the desired watermark, using both Adam and SGD to compare the results.

### 2.2. Optimization Algorithms

The training of a DNN is an iterative process that makes it possible for the model to learn how to perform a given task. This is certainly the most challenging optimization problem when implementing deep learning models from scratch. In order to increase the efficiency of the training process, researchers have developed several optimization techniques in the last years, each of them with its own advantages and drawbacks [17].

An optimization algorithm determines the weight update rule that must be applied at every iteration. The goal is usually to minimize a cost function—also known as loss function—which generally compares predictions with expected values and computes an error metric that evaluates the performance of the network. The learning rate μ—or step size—is the hyperparameter that controls how much the weights can change after each iteration of the optimization algorithm.

In the following sections, we briefly review the mechanics of two widely known optimization algorithms: SGD and Adam.

#### 2.2.1. SGD Optimization

SGD [8] is a classic optimization algorithm based on gradient descent and one of the most used. However, unlike standard gradient descent techniques that use all the training samples to compute the gradient of the cost function, SGD uses a small number of samples from the dataset—a minibatch—and then takes the average over these samples to get an estimate of the gradient, $\nabla_{w}f\left(w\right)$. Then, the update rule is given by:

(2) $$w^{\left(k\right)}=w^{\left(k-1\right)}-\mu \nabla_{w}f\left(w^{\left(k-1\right)}\right),\;\;k\geq 1$$

where $w^{\left(k\right)}$ is the weight vector at iteration *k* and $w^{\left(0\right)}$ is its initial value.

#### 2.2.2. Adam Optimization

Adam [9] is a popular optimization algorithm that combines the ideas of AdaGrad [18] and RMSProp [19]. Some of the advantages of Adam include, among others, the fact of being easy to implement and computationally efficient, as well as being fast and suitable for complex settings with noisy and sparse gradients.

Just like SGD, Adam estimates the gradient from the samples of a minibatch, but, as opposed to SGD, it uses estimates of the first and second moments of the gradients to compute individual adaptive learning rates for each parameter in the network. In order to do that, exponential moving averages of the gradient and the squared gradient are calculated using two hyperparameters to control the decay rate, $\beta_{1}$ and $\beta_{2}$, respectively. Let $m^{\left(0\right)}=0$ and $v^{\left(0\right)}=0$ be the initial values for the first and second moment vectors, respectively, then the steps of this algorithm at the *k*th iteration are the following [9]:

(3) $$\begin{array}{ccc} {\hat{g}}^{\left(k\right)} & = & \nabla_{w}f\left(w^{\left(k-1\right)}\right) \end{array}$$

(4) $$\begin{array}{ccc} m^{\left(k\right)} & = & \beta_{1}m^{\left(k-1\right)}+\left(1-\beta_{1}\right){\hat{g}}^{\left(k\right)} \end{array}$$

(5) $$\begin{array}{ccc} v^{\left(k\right)} & = & \beta_{2}v^{\left(k-1\right)}+\left(1-\beta_{2}\right)\left({\hat{g}}^{\left(k\right)}\circ {\hat{g}}^{\left(k\right)}\right) \end{array}$$

(6) $$\begin{array}{ccc} {\hat{m}}^{\left(k\right)} & = & \frac{m^{\left(k\right)}}{\left(1-\beta_{1}^{k}\right)} \end{array}$$

(7) $$\begin{array}{ccc} {\hat{v}}^{\left(k\right)} & = & \frac{v^{\left(k\right)}}{\left(1-\beta_{2}^{k}\right)} \end{array}$$

(8) $$\begin{array}{ccc} w_{j}^{\left(k\right)} & = & w_{j}^{\left(k-1\right)}-\mu \frac{{\hat{m}}_{j}^{\left(k\right)}}{\sqrt{{\hat{v}}_{j}^{\left(k\right)}}+\epsilon },\;j=1,\cdots ,N. \end{array}$$

where $w_{j}^{\left(k\right)}$, ${\hat{m}}_{j}^{\left(k\right)}$, ${\hat{v}}_{j}^{\left(k\right)}$ are the *j*th element of $w^{\left(k\right)}$, ${\hat{m}}^{\left(k\right)}$, ${\hat{v}}^{\left(k\right)}$, respectively, f(w) is the cost function and ϵ is a very small number that avoids diving by zero. The default set-up for the hyperparameters is [9]: $\beta_{1}=0.9$, $\beta_{2}=0.999$ and $\epsilon =1\cdot 10^{-8}$.

### 2.3. Digital Watermarking Algorithm

In this paper, we analyze the digital watermarking algorithm proposed in [5,6]. As we mentioned previously, we employ the fine-tune-to-embed approach; therefore, the embedding function is applied only during some additional epochs after convergence is achieved for the original task—in this case, image denoising—.

#### 2.3.1. Embedding Elements

We wish to embed a *T*-bit sequence, $b\in {\left\{0,1\right\}}^{T}$, into a certain layer *l* of the host DNN. For this specific layer, let (S,S), *I* and *F* represent the size of the convolution filter, the depth of input to the convolutional layer and the number of filters in that layer, respectively. Then, the weights can be represented by a tensor $W_{l}$ with dimensions S×S×I×F and then rearranged to form a vector $w_{l}$ of length $N=S^{2}IF$.

One important point here is that, instead of directly using the weight vector $w_{l}$, the authors in [5,6] suggest including an initial transformation of these coefficients. In order to reflect this, we must calculate the mean of $W_{l}$ over the *F* kernels. As a result, we obtain a new flattened vector ${\hat{w}}_{l}$ with *M* elements, where $M=S^{2}I$. This transformation can also be formulated if we introduce a new matrix Θ of size N×M:

$$\Theta ≐I_{M}⊗h$$

so that ${\hat{w}}_{l}=\Theta^{T}w_{l}$. Here, h is a column vector of length *F* with all of its elements set to 1/F, i.e., $h^{T}h=1/F$.

In order to move to the *T*-dimensional space, the authors in [5,6] introduce a secret projection matrix Φ. The size of this projection matrix—also referred to as regularizer parameter in [5,6]—is M×T so that each column corresponds to a particular projection vector $\phi^{\left(i\right)}$, i=1,⋯,T.

For the purpose of the subsequent theoretical analysis, we pair up matrices Φ and Θ and, therefore, we ascribe the initial transformation to the projection matrix, preserving the notation with the original weight vector $w_{l}$. To that end, we define a new N×T projection matrix:

(9) $$\hat{\Phi }=\Theta \Phi$$

so that now the projection vectors can be expressed as ${\hat{\phi }}^{\left(i\right)}=\Theta \phi^{\left(i\right)}$.

#### 2.3.2. Embedding Process

Now that we have introduced the basic elements, the embedding procedure can be described as follows [5,6]. Let w be a vector containing all the parameters of the network, then the watermarking regularizer, $f_{\mathrm{watermark}}\left(w_{l}\right)$, is added to the global cost function f(w):

$$f\left(w\right)=f_{0}\left(w\right)+\lambda f_{\mathrm{watermark}}\left(w_{l}\right)$$

where $f_{0}\left(w\right)$ is the original cost function and λ is the regularization parameter. The regularizer term is a composition of two functions: cross-entropy and the sigmoid,

(10) $$f_{\mathrm{watermark}}\left(w_{l}\right)≐-\sum_{i=1}^{T}\left(b_{i}\log\left(y_{i}\right)+\left(1-b_{i}\right)\log\left(1-y_{i}\right)\right)$$

with $y_{i}=1/\left(1+\exp(-{\left({\hat{\phi }}^{\left(i\right)}\right)}^{T}w_{l})\right)$. In order to minimize (10), $y_{i}$ will approximate to the value of $b_{i}$. Therefore, the sigmoid function will force each projection ${\left({\hat{\phi }}^{\left(i\right)}\right)}^{T}w_{l}$, i=1,⋯,T, to progressively move towards +∞ and −∞ depending on whether $b_{i}=1$ or $b_{i}=0$, respectively. For successfully embedding the secret message, it is generally enough to guarantee that each projection lies on the proper side of the horizontal axis. When this happens, we reach a Bit Error Rate (BER) of 0 and all the projected weights are aligned with their corresponding bits, that is, they are positive when the bit is 1 and, conversely, negative when the bit is 0.

#### 2.3.3. Detectability Issues

In this paper, we employ the Random technique proposed by the authors, in which the values of the projection matrix Φ—before applying the transformation in (9)—are independent samples from a standard normal distribution N(0,1). Their results [5,6] show that this Random approach is the most appealing design method for Φ because, as they indicate, it does not significantly alter the distribution of weights at the embedding layer. However, there are some detectability issues here that should be considered.

On the one hand, the authors in [20] show that the standard deviation of the distribution of weights grows with the length of the embedded message. This information can be used by adversaries for detecting the watermark and even overwriting it.

On the other hand, one of the main conclusions of our work is that the presence or absence of alterations to the shape of the weight distributions is a consequence of the optimization algorithm used during the watermark embedding. In particular, the authors in [5,6] employ SGD with momentum in their experiments and the distributions of weights remain unchanged, yet the use of Adam would significantly alter the shape of the distributions even though we apply the same Random technique. In this paper, we will show that the results in [5,6] regarding the undetectability of the watermark do not hold when we use Adam optimization.

**Figure 2:**
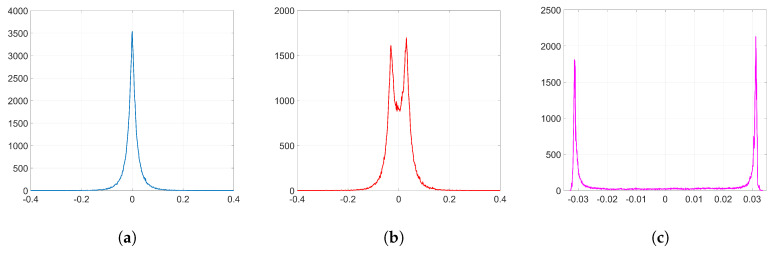
Histograms from the embedding layer l=2 (T=256, λ=1 and *k* = 32,140). (**a**) histogram of $w^{\left(0\right)}$; (**b**) histogram of $w^{\left(k\right)}$; (**c**) histogram of $\Delta w=w^{\left(k\right)}-w^{\left(0\right)}$.

As an example to visualize this peculiar behavior shown by Adam when we employ the watermarking algorithm proposed in [5,6], we plot in Figure 2 the resulting histograms when T=256 and λ=1; specifically, the histograms of the weights before and after the embedding (corresponding to *k* = 32,140) and the histogram of the weight variations, respectively. As we can see, the distribution of the original weights has significantly changed, turning into a two-spiked shape that could be easily detected by an adversary. The complete set of histograms will be later shown in Section 6.

#### 2.3.4. Gaussian and Orthogonal Projection Vectors

In addition to the Random technique suggested by the authors of this watermarking algorithm—whose projection vectors will be referred to as Gaussian projection vectors in this paper—we will also implement orthonormal projectors. In order to build these kinds of projectors, we first generate the projection matrix following the Random technique; that is, samples are drawn from a standard normal distribution N(0,1). Then, from the Singular Value Decomposition of this projection matrix, we obtain an orthonormal basis for the column space so that we have $\Phi^{T}\Phi =I_{T}$. Notice that once we apply the initial transformation (i.e., $\hat{\Phi }=\Theta \Phi$) the resulting projection vectors ${\hat{\phi }}^{\left(i\right)}$, i=1,⋯,T, will still preserve the orthogonality between them, although they will not be normalized:

$${\left(\hat{\Phi }\right)}^{T}\hat{\Phi }={\left(\Theta \Phi \right)}^{T}\Theta \Phi =\Phi^{T}\Theta^{T}\Theta \Phi =\Phi^{T}\left(I_{M}⊗h^{T}\right)\left(I_{M}⊗h\right)\Phi =\frac{1}{F}\Phi^{T}\Phi =\frac{1}{F}I_{T}$$

Therefore, these kinds of projectors will be referred to as orthogonal. As we will see later from KLD results and histograms, implementing orthogonal projectors may help us to better preserve both the original shape of the weight distribution and the denoising performance.

## 3. Theoretical Analysis

From now on, we will omit the sub-index *l* for the sake of clarity although we are always addressing the coefficients of the embedding layer. The experiments clearly illustrate that the use of Adam optimization together with the watermarking algorithm proposed in [5,6] originates noticeable changes in the distribution of weights, as we see in Figure 2. In the following analysis, we delve into the reasons why this happens. To that end, we aim to get a theoretical expression of $\Delta w=w^{\left(k\right)}-w^{\left(0\right)}$. This will allow us to prove and understand the nature of the observed behavior of the weights when watermark embedding is carried out. We start off by defining vector $\hat{\varphi }$ and matrix $\hat{\Psi }$:

(11) $$\begin{array}{ccc} \hat{\varphi } & ≐ & \sum_{i=1}^{T}{\hat{\phi }}^{\left(i\right)} \end{array}$$

(12) $$\begin{array}{ccc} \hat{\Psi } & ≐ & \sum_{i=1}^{T}{\hat{\phi }}^{\left(i\right)}{\left({\hat{\phi }}^{\left(i\right)}\right)}^{T} \end{array}$$

Notice that $\hat{\Psi }={\hat{\Psi }}^{T}$. In addition, for the case of orthogonal projectors, the following properties can be straightforwardly proven:

(13) $$\begin{array}{ccc} \hat{\Psi }\hat{\varphi } & = & \frac{1}{F}\hat{\varphi } \end{array}$$

(14) $$\begin{array}{ccc} \hat{\Psi }\hat{\Psi } & = & \frac{1}{F}\hat{\Psi } \end{array}$$

These properties will come in handy later on in several theoretical derivations.

Firstly, in order to simplify the analysis and understand more clearly how the watermarking cost function impacts on the movement of the weights when using both SGD and Adam optimization, we will just consider the presence of the regularization term, that is, we will not include the denoising cost function for now. The influence of the denoising part will be studied in Section 3.3. Therefore, given our embedding cost function in (10) and assuming for simplicity (and without loss of generality) that all embedded symbols are +1, we have:

$$\begin{array}{c} \tilde{f}\left(w\right)≐\lambda f_{\mathrm{watermark}}\left(w|b=1\right)=\lambda \sum_{i=1}^{T}\log\left(1+\exp\left(-{\left({\hat{\phi }}^{\left(i\right)}\right)}^{T}w\right)\right) \end{array}$$

If we compute the gradient of this function, we obtain:

(15) $$\nabla_{w}\tilde{f}\left(w\right)=-\lambda \sum_{i=1}^{T}{\hat{\phi }}^{\left(i\right)}\frac{1}{1+\exp\left({\left({\hat{\phi }}^{\left(i\right)}\right)}^{T}w\right)}$$

In order to simplify the subsequent analysis, we introduce a series of assumptions which are based on empirical observations or hypotheses that will be duly verified.

By construction, it is possible to show that the mean of ${\left({\hat{\phi }}^{\left(i\right)}\right)}^{T}w^{\left(0\right)}$ is zero and its variance is $\left(N/F^{2}\right)\mathrm{Var}\left\{w^{\left(0\right)}\right\}$ for Gaussian projectors and $\left(1/F\right)\mathrm{Var}\left\{w^{\left(0\right)}\right\}$ for orthogonal projectors (see Appendix A.1). Since the variance of the weights at the initial iteration is generally very small—in our experiments, it is 0.0012—it can be considered that the variance of ${\left({\hat{\phi }}^{\left(i\right)}\right)}^{T}w^{\left(0\right)}$ will also be small enough so that we can assume $|{\left({\hat{\phi }}^{\left(i\right)}\right)}^{T}w|\ll 1$ for all i=1,⋯,T. Although this assumption might not be strictly true for all *k*—especially once we have crossed the linear region of the sigmoid function—it is reasonably good and it allows us to use a first-order Taylor expansion for ${\left(1+\exp\left({\left({\hat{\phi }}^{\left(i\right)}\right)}^{T}w\right)\right)}^{-1}$ around ${\left({\hat{\phi }}^{\left(i\right)}\right)}^{T}w=0$:

(16) $${\left(1+\exp\left({\left({\hat{\phi }}^{\left(i\right)}\right)}^{T}w\right)\right)}^{-1}\approx \frac{1}{2}-\frac{{\left({\hat{\phi }}^{\left(i\right)}\right)}^{T}w}{4}$$

Plugging (16) into (15) and using the definitions given above, we can write:

(17) $$\nabla_{w}\tilde{f}\left(w\right)\approx -\frac{\lambda }{2}\hat{\varphi }+\frac{\lambda }{4}\hat{\Psi }w$$

We introduce now one important hypothesis in this theoretical analysis to handle the previous equation: we assume that $w^{\left(k\right)}$ grows approximately affinely with *k*:

(18) $$w^{\left(k\right)}\approx w^{\left(0\right)}+k\cdot \mu \cdot \eta$$

where η is a vector that contains the slopes for each weight, and it is to be determined in the following sections. We hypothesize this affine-like growth for the weights and, later, we will verify that this is consistent with the rest of the theory and the experiments (see Appendix B.1 for more details). Therefore, we can write the weight variations as:

(19) $$\Delta w=w^{\left(k\right)}-w^{\left(0\right)}\approx k\cdot \mu \cdot \eta$$

### 3.1. Analysis for SGD

We first analyze the behavior of SGD optimization when we implement digital watermarking as proposed in [5,6]. Recall the SGD update rule in (2). If we use the approximation for the gradient in (17) and the affine growth hypothesis for the weights introduced in (18), we have:

(20) $$\nabla_{w}\tilde{f}\left(w\right)\approx -\frac{\lambda }{2}\hat{\varphi }+\frac{\lambda }{4}\hat{\Psi }\left(w^{\left(0\right)}+k\mu \eta \right)$$

To simplify the analysis, we consider from now on that $w^{\left(0\right)}\approx 0$. We confirm the validity of this assumption in Appendix B.2. Then, we can write:

(21) $$\eta =-\nabla_{w}\tilde{f}\left(w\right)\approx \frac{\lambda }{2}\hat{\varphi }-\frac{\lambda k\mu }{4}\hat{\Psi }\eta$$

If we consider orthogonal projectors, we can arrive at a more explicit expression for η. In particular, if we multiply (21) by $\hat{\Psi }$ and use the properties (13) and (14), we obtain:

(22) $$\hat{\Psi }\eta =\frac{2\lambda }{4F-\lambda k\mu }\hat{\varphi }$$

Then, substituting (22) into (21), we can get a more concise expression for η:

(23) $$\eta \approx \frac{\lambda }{2}\left(1-\frac{\lambda k\mu }{4F-\lambda k\mu }\right)\hat{\varphi }$$

Thus, if *F* is large compared to λkμ—this certainly holds for our experimental set-up, cf. Section 6.1—η will be approximately proportional to $\hat{\varphi }$. Then, the coefficients will follow an affine-like growth as we hypothesized in (18) (see Appendix B.1 for the empirical confirmation of this hypothesis). Now, the weight variations can be expressed as:

(24) $$\Delta w\approx k\cdot \mu \cdot \eta =\frac{\lambda k\mu }{2}\left(1-\frac{\lambda k\mu }{4F-\lambda k\mu }\right)\hat{\varphi }$$

As we can see, when we use SGD, Δw will approximately follow a zero-mean Gaussian distribution, as induced by [9]. Because of this, and unlike Adam (as we will see later), the weights will evolve with random speeds when we embed watermarks using SGD optimization. Therefore, the impact on the original shape of the weight distribution will be small. However, the variance of the weight distribution may change considerably as stated in [20]. Since we have $\mathrm{Var}\left\{\hat{\varphi }\right\}=T/\left(FN\right)$ for orthogonal projectors, the variance of Δw can be computed as:

$$\mathrm{Var}\left\{\Delta w\right\}={\left[\lambda k\mu \frac{\left(2F-\lambda k\mu \right)}{\left(4F-\lambda k\mu \right)}\right]}^{2}\frac{T}{FN}$$

Thus, considering that $w^{\left(0\right)}$ and Δw are uncorrelated—we check this statement in Section 6.2.2—we arrive at the following expression for the variance of the weights at the *k*th iteration:

(25) $$\mathrm{Var}\left\{w^{\left(k\right)}\right\}=\mathrm{Var}\left\{w^{\left(0\right)}\right\}+{\left[\lambda k\mu \frac{\left(2F-\lambda k\mu \right)}{\left(4F-\lambda k\mu \right)}\right]}^{2}\frac{T}{FN}$$

As we can see from (25), when implementing the digital watermarking algorithm in [5,6] with SGD optimization and orthogonal projectors, the variance of the resulting weight distribution might change considerably. In order to preserve the original weight distribution when using SGD, it is important to take care with the values of *T*, *F* and *N*, especially. In addition, the standard deviation of the weights will (approximately) increase linearly with the number of iterations so it may be also important to limit the value of *k*. This is in line with the expected behavior: the weights will move away from their original value and they will be further if we perform more iterations.

Because the analysis for Gaussian projectors becomes considerably difficult, in this paper, we just address the study of SGD with orthogonal projectors. A more comprehensive analysis for Gaussian projectors that can be linked to the results obtained in [20] is left for future research. Regardless of this, the whole analysis for both kinds of projection vectors will be developed in the next section for Adam optimization.

### 3.2. Analysis for Adam

In the next sections, we will delve into the theory behind Adam optimization for DNN watermarking. In particular, we will obtain an expression for the mean and the variance of the gradient and then, as we did with SGD, we will analyze the update term to get an expression of the weight variations.

#### 3.2.1. Mean of the Gradient

We are interested in computing the mean of the gradient that is used in Adam. Considering $\tilde{f}\left(w\right)$ as the global cost function, then, from (4), we can rewrite the mean at the *k*th iteration as:

(26) $$m^{\left(k\right)}=\left(1-\beta_{1}\right)\sum_{i=1}^{k}\beta_{1}^{k-i}\nabla_{w}\tilde{f}\left(w^{\left(i\right)}\right)$$

We use the gradient in (17) and do some derivations to find an explicit expression for ${\hat{m}}^{\left(k\right)}=m^{\left(k\right)}/\left(1-\beta_{1}^{k}\right)$ under the hypothesis in (18). Finally, we arrive at the following expression for the bias-corrected mean gradient when *k* is sufficiently large (see Appendix A.2 for all the mathematical details):

(27) $$\begin{array}{ccc} {\hat{m}}^{\left(k\right)} & \approx & -\frac{\lambda }{2}\hat{\varphi }+\frac{\lambda }{4}\cdot \hat{\Psi }\left(w^{\left(0\right)}+k\mu \eta \right)\\ & = & \frac{\lambda }{2}\left(m_{0}+\mu \cdot k\cdot m_{1}\right),\;\;k\gg \frac{\beta_{1}}{1-\beta_{1}} \end{array}$$

where $m_{0}≐-\hat{\varphi }+\frac{1}{2}\hat{\Psi }w^{\left(0\right)}$ and $m_{1}≐\frac{1}{2}\hat{\Psi }\eta$. As we see from (27), the mean of the gradient also grows affinely with *k*.

#### 3.2.2. Variance of the Gradient

Let $g\left(w\right)≐\nabla_{w}\tilde{f}\left(w\right)\circ \nabla_{w}\tilde{f}\left(w\right)$. The approximation in (17) for the *j*th element of this vector g(w), denoted by $g_{j}\left(w\right)$, is the following:

$$\begin{array}{ccc} g_{j}\left(w\right) & \approx & \frac{\lambda^{2}}{4}\sum_{m=1}^{T}\sum_{l=1}^{T}{\hat{\phi }}_{j}^{\left(m\right)}{\hat{\phi }}_{j}^{\left(l\right)}\left(1-{\left({\hat{\phi }}^{\left(m\right)}\right)}^{T}w+\frac{1}{4}{\left({\hat{\phi }}^{\left(m\right)}\right)}^{T}ww^{T}{\hat{\phi }}^{\left(l\right)}\right)\\ & = & \frac{\lambda^{2}}{4}\left(a_{j}-b_{j}^{T}w+\frac{1}{4}c_{j}^{T}ww^{T}c_{j}\right) \end{array}$$

where:

(28) $$\begin{array}{ccc} a_{j} & ≐ & {\hat{\varphi }}_{j}^{2} \end{array}$$

(29) $$\begin{array}{ccc} c_{j} & ≐ & \sum_{m=1}^{T}{\hat{\phi }}_{j}^{\left(m\right)}{\hat{\phi }}^{\left(m\right)} \end{array}$$

(30) $$\begin{array}{ccc} b_{j} & ≐ & {\hat{\varphi }}_{j}\cdot c_{j} \end{array}$$

Following the hypothesis in (18), we can write:

$$\begin{array}{c} g_{j}\left(w^{\left(k\right)}\right)\approx \frac{\lambda^{2}}{4}\left(a_{j}-b_{j}^{T}w^{\left(0\right)}+\frac{1}{4}c_{j}^{T}w^{\left(0\right)}{\left(w^{\left(0\right)}\right)}^{T}c_{j}-k\mu b_{j}^{T}\eta +\frac{1}{2}k\mu c_{j}^{T}w^{\left(0\right)}\eta^{T}c_{j}+\frac{1}{4}k^{2}\mu^{2}c_{j}^{T}\eta \eta^{T}c_{j}\right) \end{array}$$

In summary, for this affine-like growth, the square gradient vector can be written as:

(31) $$g_{j}\left(w^{\left(k\right)}\right)\approx \frac{\lambda^{2}}{4}\left(p_{j}+q_{j}k\mu +r_{j}k^{2}\mu^{2}\right)$$

for some vectors p,q,r whose *j*th component can be defined as:

(32) $$\begin{array}{ccc} p_{j} & ≐ & a_{j}-b_{j}^{T}w^{\left(0\right)}+\frac{1}{4}c_{j}^{T}w^{\left(0\right)}{\left(w^{\left(0\right)}\right)}^{T}c_{j}\\ q_{j} & ≐ & -b_{j}^{T}\eta +\frac{1}{2}c_{j}^{T}w^{\left(0\right)}\eta^{T}c_{j} \end{array}$$

(33) $$\begin{array}{ccc} r_{j} & ≐ & \frac{1}{4}c_{j}^{T}\eta \eta^{T}c_{j} \end{array}$$

Now, from (5), we can rewrite the variance of the gradient that is used in Adam as:

(34) $$v_{j}^{\left(k\right)}=\left(1-\beta_{2}\right)\sum_{i=1}^{k}\beta_{2}^{k-i}g_{j}\left(w^{\left(i\right)}\right)$$

The bias-corrected term ${\hat{v}}_{j}^{\left(k\right)}$ is obtained after dividing $v_{j}^{\left(k\right)}$ by $\left(1-\beta_{2}^{k}\right)$. Applied to the special case of (31), this yields (see Appendix A.3):

$$\begin{array}{ccc} {\hat{v}}_{j}^{\left(k\right)} & = & \frac{\lambda^{2}}{4}\left(p_{j}-\frac{\beta_{2}}{1-\beta_{2}}\mu q_{j}+\frac{\beta_{2}+\beta_{2}^{2}}{{\left(1-\beta_{2}\right)}^{2}}\mu^{2}r_{j}\right)\\ & + & \frac{\lambda^{2}}{4(1-\beta_{2}^{k})}k\left(\mu q_{j}-\frac{2\beta_{2}}{1-\beta_{2}}\mu^{2}r_{j}\right)+\frac{\lambda^{2}}{4(1-\beta_{2}^{k})}k^{2}\mu^{2}r_{j} \end{array}$$

#### 3.2.3. Update Term

Because μ is usually very small—we use $\mu =1\cdot 10^{-6}$ in our experiments—we can assume that kμ will be small enough to obtain an approximation of the update used in Adam. Recall that, for the *j*th weight, this is $u_{j}^{\left(k\right)}≐{\hat{m}}_{j}^{\left(k\right)}/\left(\sqrt{{\hat{v}}_{j}^{\left(k\right)}}+\epsilon \right)$, implying that $w_{j}^{\left(k\right)}=w_{j}^{\left(k-1\right)}-\mu u_{j}^{\left(k\right)}$. Let:

(35) $$s_{j}≐\left(p_{j}-\frac{\beta_{2}}{1-\beta_{2}}\mu q_{j}+\frac{\beta_{2}+\beta_{2}^{2}}{{\left(1-\beta_{2}\right)}^{2}}\mu^{2}r_{j}\right)$$

Then, assuming that μk≪1, we can make a zero-order approximation of the update term, i.e.,:

(36) $$u_{j}^{\left(k\right)}=\frac{{\hat{m}}_{j}^{\left(k\right)}}{\sqrt{{\hat{v}}_{j}^{\left(k\right)}}+\epsilon }\approx \frac{m_{0,j}}{\sqrt{s_{j}}},\;\;\mu k\ll 1$$

This approximation is accurate enough for the set of experiments we perform. In particular, for the orthogonal case, we could deal with $k_{max}$ = 625,000 and still get a correlation coefficient of 0.9900 between $\left(\lambda^{2}/4\right)s_{j}$ and ${\hat{v}}_{j}^{\left(k_{max}\right)}$. In our experiments, we actually reach a BER of zero for values of *k* quite below $k_{max}$ (cf. Section 6.2).

From (36), we observe that the updated *j*th coefficient approximately follows the hypothesized growth, i.e., $w^{\left(k\right)}=w^{\left(0\right)}+k\cdot \mu \cdot \eta$, where $\eta_{j}=-m_{0,j}/\sqrt{s_{j}}$. Notice that, as expected, the update does not depend on λ, following Adam’s property that the update is invariant to rescaling the gradients [9]. Finding a more explicit expression runs into the problem that η depends on s, which in turn is a function of η through (32) and (33). The following subsections are devoted to solving this problem by conjecturing a form for η and refining it.

To simplify the analysis, we consider from now on that $w^{\left(0\right)}\approx 0$ since most of the values of the weights at the initial iteration are very small (see Figure 2a). We will verify the accuracy of this approximation in Appendix B.2.

#### 3.2.4. Rationale for the Sign Function

Recall the expression (23) that we obtained for η when analyzing SGD, where we found η to be approximately proportional to $\hat{\varphi }$. Now, for Adam, we take this as a starting point, so we conjecture first that $\eta =\gamma \cdot \hat{\varphi }$, for some real positive γ. Here, we consider orthogonal projection vectors and use the property introduced in (13) and the following:

$$c_{j}^{T}\hat{\varphi }=\frac{{\hat{\varphi }}_{j}}{F}$$

In this particular case, we have the following identities:

$$\begin{array}{ccc} m_{0,j} & \approx & -{\hat{\varphi }}_{j}\\ p_{j} & \approx & a_{j}={\hat{\varphi }}_{j}^{2}\\ q_{j} & \approx & -\frac{\gamma }{F}{\hat{\varphi }}_{j}^{2}\\ r_{j} & = & \frac{\gamma^{2}}{4F^{2}}{\hat{\varphi }}_{j}^{2} \end{array}$$

Substituting these values into (35), we find that

$$\sqrt{s_{j}}=\mathrm{sgn}\left({\hat{\varphi }}_{j}\right)\cdot {\hat{\varphi }}_{j}\cdot \sqrt{1+\left(\gamma /F\right)\mu \beta_{2}/\left(1-\beta_{2}\right)+\left(\gamma^{2}/\left(4F^{2}\right)\right)\mu^{2}\left(\beta_{2}+\beta_{2}^{2}\right)/{\left(1-\beta_{2}\right)}^{2}}$$

When we divide $m_{0,j}$ by $\sqrt{s_{j}}$, we obtain:

(37) $$u_{j}^{\left(k\right)}\approx \frac{-\mathrm{sgn}({\hat{\varphi }}_{j})}{\sqrt{1+\left(\gamma /F\right)\mu \beta_{2}/\left(1-\beta_{2}\right)+\left(\gamma^{2}/\left(4F^{2}\right)\right)\mu^{2}\left(\beta_{2}+\beta_{2}^{2}\right)/{\left(1-\beta_{2}\right)}^{2}}}$$

It is then clear that η cannot be written in the form $\eta =\gamma \cdot \hat{\varphi }$, as was conjectured at the beginning of this section.

#### 3.2.5. A Theoretical Expression for Δw

Although the conjectured form for η in Section 3.2.4 does not hold, the appearance of the sign function in (37) gives a key clue for an alternative approach, since the sign seems to reveal the reason behind the two-spiked histograms like the one shown in Figure 2c.

Therefore, let us write $\eta_{j}$ to explicitly contain the sign of $\varphi_{j}$ and allow $\gamma_{j}$ to take different (non-negative) values with *j* to reflect the varying magnitude (recall that even in Section 3.2.4 the conjectured value could be written as $\eta_{j}=\gamma \left|\varphi_{j}\right|\cdot \mathrm{sgn}\left(\varphi_{j}\right)$). Let γ be the column vector containing $\gamma_{j}$, j=1,⋯,N, then $\eta =\gamma \circ \mathrm{sgn}(\hat{\varphi })$. Since $c_{j}^{T}\left(\gamma \circ \mathrm{sgn}\left(\hat{\varphi }\right)\right)=\gamma^{T}\left(c_{j}\circ \mathrm{sgn}\left(\hat{\varphi }\right)\right)$, we can write:

$$\begin{array}{ccc} m_{0,j} & \approx & -{\hat{\varphi }}_{j}\\ p_{j} & \approx & a_{j}={\hat{\varphi }}_{j}^{2}\\ q_{j} & \approx & -b_{j}^{T}\eta =-{\hat{\varphi }}_{j}\gamma^{T}\left(c_{j}\circ \mathrm{sgn}\left(\hat{\varphi }\right)\right)\\ r_{j} & = & \frac{1}{4}{\left[\gamma^{T}\left(c_{j}\circ \mathrm{sgn}\left(\hat{\varphi }\right)\right)\right]}^{2} \end{array}$$

Now,

$$u_{j}^{\left(k\right)}\approx \frac{-{\hat{\varphi }}_{j}}{\sqrt{{\hat{\varphi }}_{j}^{2}+\mu {\hat{\varphi }}_{j}\gamma^{T}\left(c_{j}\circ \mathrm{sgn}\left(\hat{\varphi }\right)\right)\frac{\beta_{2}}{\left(1-\beta_{2}\right)}+\frac{\mu^{2}}{4}{\left[\gamma^{T}\left(c_{j}\circ \mathrm{sgn}\left(\hat{\varphi }\right)\right)\right]}^{2}\frac{\beta_{2}+\beta_{2}^{2}}{{\left(1-\beta_{2}\right)}^{2}}}}$$

In addition, thus, in order to meet the condition $\eta_{j}=\gamma_{j}\cdot \mathrm{sgn}\left({\hat{\varphi }}_{j}\right)$, the following nonlinear equation should be solved for all $\gamma_{j}$, j=1,⋯,N:

(38) $$\gamma_{j}=\frac{|{\hat{\varphi }}_{j}|}{\sqrt{{\hat{\varphi }}_{j}^{2}+\mu {\hat{\varphi }}_{j}\gamma^{T}\left(c_{j}\circ \mathrm{sgn}\left(\hat{\varphi }\right)\right)\frac{\beta_{2}}{\left(1-\beta_{2}\right)}+\frac{\mu^{2}}{4}{\left[\gamma^{T}(c_{j}\circ \mathrm{sgn}\left(\hat{\varphi }\right)\right]}^{2}\frac{\beta_{2}+\beta_{2}^{2}}{{\left(1-\beta_{2}\right)}^{2}}}}$$

This equation can be solved with a fixed-point iteration method [21]. To that end, we should initialize γ and then iterate the following: (1) compute the right-hand side of (38), and (2) use it to update γ on the left-hand side. This process will converge to the solution of (38). Even though this method can be implemented to give the specific values for each $\gamma_{j}$, we are more interested in obtaining a statistical characterization rather than a deterministic one. As we will see, the statistical approach offers a deeper explanation for the two-spiked distribution of Δw which we ultimately seek.

We thus aim at finding the pdf of Γ, now considered as a random variable for which $\gamma_{j}$, j=1,⋯,N are nothing but realizations. Once again, Equation (38) can be solved iteratively (e.g., with Markov-chain Monte Carlo methods [22]) to yield the equilibrium distribution for Γ. Instead, we can resort to the results in Section 6 where we conclude that the pdf of Γ is strongly concentrated around its mode. With this observation, it is possible to consider that $\gamma^{T}\left(c_{j}\circ \mathrm{sgn}\left(\hat{\varphi }\right)\right)$ approximately corresponds to realizations of $\Gamma \cdot (c_{j}^{T}\mathrm{sgn}\left(\hat{\varphi }\right))$.

In order to simplify the analysis even further, we are interested in decomposing $c_{j}^{T}\mathrm{sgn}\left(\hat{\varphi }\right)$ using its statistical projection onto ${\hat{\varphi }}_{j}$, i.e., $c_{j}^{T}\mathrm{sgn}\left(\hat{\varphi }\right)=\alpha \cdot {\hat{\varphi }}_{j}+z_{j}$. Here, α is a real multiplier and $z_{j}$ is zero-mean noise uncorrelated with ${\hat{\varphi }}_{j}$. More generally, if we define matrix $C≐[c_{0},\cdots ,c_{N}]$, then we seek to write $C^{T}\mathrm{sgn}\left(\hat{\varphi }\right)=\alpha \cdot \hat{\varphi }+z$. We do the analysis for the cases of Gaussian projectors and orthogonal projectors separately (refer to Appendix A.4 for the derivations). For the Gaussian case, we get:

(39) $$\begin{array}{ccc} \alpha & = & \frac{1}{FT}\sqrt{\frac{2}{\pi T}}\left(FT^{2}+\left(N+F\right)T-F\right) \end{array}$$

(40) $$\begin{array}{ccc} \mathrm{Var}(z_{j}) & = & -\frac{2}{\pi F^{3}T^{2}}\left(FT^{4}+4FT^{3}+\left(N+F\right)T^{2}-4FT+F\right)+\frac{T}{F^{3}}\left(N+F\left(T+1\right)\right) \end{array}$$

On the other hand, for the orthogonal projectors, we get instead:

(41) $$\begin{array}{ccc} \alpha & = & \sqrt{\frac{2N}{\pi TF}} \end{array}$$

(42) $$\begin{array}{ccc} \mathrm{Var}(z_{j}) & = & \left(1-\frac{2}{\pi }\right)\frac{T}{FN} \end{array}$$

Recall that, by construction, $\hat{\varphi }$ can be seen as a random vector. In fact, we have $\hat{\varphi }∼N\left(0,I\cdot T/F^{2}\right)$ for Gaussian projection vectors, and $\hat{\varphi }$ approximately follows N(0,I·T/(FN)) for orthogonal projectors. Let Ξ, *Z* be random variables with the distribution of a single element of $\hat{\varphi }$ and z, respectively, then $q_{j}$ and $r_{j}$ can be seen as realizations of (approximately): Q=−ΓΞ(αΞ+Z) and $R=\frac{\Gamma^{2}}{4}{\left(\alpha \Xi +Z\right)}^{2}$, so a stochastic version of (38) is:

$$\Gamma =\frac{\left|\Xi \right|}{\sqrt{\Xi^{2}+\Gamma \mu \Xi \left(\alpha \Xi +Z\right)\frac{\beta_{2}}{\left(1-\beta_{2}\right)}+\frac{\Gamma^{2}\mu^{2}}{4}{\left(\alpha \Xi +Z\right)}^{2}\frac{\beta_{2}+\beta_{2}^{2}}{{\left(1-\beta_{2}\right)}^{2}}}}$$

Squaring both sides, we find that, for a given realization (ξ, *z*) of (Ξ, *Z*), Γ must take the positive value γ that satisfies the following fourth degree equation:

(43) $$\frac{\beta_{2}+\beta_{2}^{2}}{{\left(1-\beta_{2}\right)}^{2}}\frac{\mu^{2}}{4}{\left(\alpha \xi +z\right)}^{2}\gamma^{4}+\frac{\beta_{2}}{1-\beta_{2}}\mu \xi \left(\alpha \xi +z\right)\gamma^{3}+\xi^{2}\gamma^{2}-\xi^{2}=0$$

From (43), it is easy to generate samples γ of Γ and, accordingly, samples of Δw, by recalling that:

(44) Δw≈k·μ·γ·sgn(ξ)

We note that, for the particular case when $\beta_{2}$ is very close to 1, $\frac{\beta_{2}+\beta_{2}^{2}}{{\left(1-\beta_{2}\right)}^{2}}\approx 2{\left(\frac{\beta_{2}}{1-\beta_{2}}\right)}^{2}$. This simplification allows us to approximate (43) as

(45) $$\gamma^{2}{\left(\frac{\gamma \mu }{2}\cdot \frac{\beta_{2}}{1-\beta_{2}}\left(\alpha \xi +z\right)+\xi \right)}^{2}-\xi^{2}\approx 0$$

which leads to the following fixed-point equation:

(46) $$\gamma \approx \frac{\xi }{\frac{\gamma \mu }{2}\cdot \frac{\beta_{2}}{1-\beta_{2}}\left(\alpha \xi +z\right)+\xi }$$

When the noise term *z* is very small compared to αξ (which occurs with a fairly large probability, especially for the case of orthogonal projectors), then the solution to (46), denoted by $\gamma_{s}$, will be independent of the value of ξ. This will cause the probability of Γ to be concentrated around $\gamma_{s}$, and in turn this will make the pdf Δw have two spikes centered at $\pm k\mu \gamma_{s}$. We will see these spikes appearing time and again in the experiments carried out with Adam (Section 6).

### 3.3. The Denoising Term

Thus far, we have considered only that our cost function is $\tilde{f}\left(w\right)=\lambda f_{\mathrm{watermark}}\left(w\right)$; however, as we know, there is an additional term, the original denoising function, so our real cost function is: $f\left(w\right)=f_{0}\left(w\right)+\tilde{f}\left(w\right)$.

The gradients corresponding to this function, $f_{0}\left(w\right)$, will try to pull the weight vector towards the original optimal $w^{\left(0\right)}$ in a relatively hard to model way. In order to analyze this behavior, we can approximate the gradient of the denoising function at the *k*th iteration with respect to the *j*th coefficient as a sum of a constant term, $d_{j}$, and a noisy one, ${\tilde{n}}_{j}^{\left(k\right)}$, which follows a zero-mean Gaussian distribution and is associated with the use of different training batches on each step. We will refer to this noise as batching noise. Thus, for each coefficient *j*, we can write:

(47) $$\frac{\partial f_{0}\left(w^{\left(k\right)}\right)}{\partial w_{j}}\approx d_{j}+{\tilde{n}}_{j}^{\left(k\right)}$$

Like we did in the previous section, we can formulate a stochastic version of (47). To that end, we notice that the constant term of this gradient, $d_{j}$, can take different values with *j*, as well as the variance of the batching noise, $h_{j}$ that is, ${\tilde{n}}_{j}$ is drawn from $N(0,h_{j})$. Therefore, in order to reflect the variability of these terms along the *j*-elements, we introduce two random variables with the distribution of the mean gradient and the variance of the batching noise, *D* and *H*, respectively, for which $d_{j}$ and $h_{j}$ are realizations. The pdf of these distributions will be obtained empirically in Section 6.2. Then, we can see ${\tilde{n}}_{j}^{\left(k\right)}$ as a realization of $\tilde{N}∼N\left(0,H\right)$.

#### 3.3.1. SGD

Similarly to Section 3.2.5, let Ξ be a random variable with the distribution of $\hat{\varphi }$. Let (ξ, δ, $\tilde{n}$) be a realization of (Ξ, *D*, $\tilde{N}$), respectively, then for SGD using orthogonal projectors we can compute samples of Δw adding both functions, i.e., denoising and watermarking:

(48) $$\Delta w\approx k\mu \left(\delta +\tilde{n}\right)+\frac{\lambda k\mu }{2}\left(1-\frac{\lambda k\mu }{4F-\lambda k\mu }\right)\xi$$

#### 3.3.2. Adam

The variance of the batching noise computed by Adam will be approximately given by the random variable *V*, whose realizations can be expressed as $v_{j}=\frac{1-\beta_{2}}{1-\beta_{2}^{k}}\sum_{i=1}^{k}\beta_{2}^{\left(k-i\right)}{\left({\tilde{n}}_{j}^{\left(i\right)}\right)}^{2}$. Notice that, for each realization of *V*, as the sum takes places over *i*, we must work with a fixed value $h_{j}$ for the variance of the batching noise. Then, with this variance, we generate *k* samples of $\tilde{N}$ to be used in the sum that produces $v_{j}$. With this characterization, we can easily analyze how the denoising cost function shapes the distribution of the weight variations. Notice that this analysis could be adapted for any host network. Let δ and ν be realizations of *D* and *V*, respectively, then, we can generate samples of Δw without including the gradients from the watermarking function as:

$$\Delta w\approx k\cdot \mu \cdot \frac{\delta }{\sqrt{\delta^{2}+\nu }}$$

Moreover, in order to get a more accurate description of the problem, we can combine both functions: denoising and watermarking. The analysis becomes somewhat complicated, but, as we will check in Section 6, the distributions resulting from this analysis do capture better the shapes observed in the empirical ones. See Appendix A.5 for the results of this analysis.

## 4. Block-Orthonormal Projections (BOP)

Here, we discuss BOP, the solution we propose to solve the detectability problem posed by Adam optimization when implementing the watermarking algorithm proposed in [5,6]. In order to hide the noticeable weight variations that appear when we use Adam—as seen in Figure 2—we introduce a prior transformation using a secret N×N matrix X (the details for its construction are given below). The procedure we follow has three steps per each iteration of Adam.

Firstly, we project the weights and gradients from the embedding layer using X:

$$\begin{array}{ccc} y & = & Xw\\ \nabla_{y}f\left(y\right) & = & X\nabla_{w}f\left(w\right) \end{array}$$

Then, we run Adam optimization on the projected weights, y, using the projected gradients, $\nabla_{y}f\left(y\right)$, as well, i.e., steps (3)–(8) are taken using y and $\nabla_{y}f\left(y^{\left(k-1\right)}\right)$ instead of w and $\nabla_{w}f\left(w^{\left(k-1\right)}\right)$, respectively. The key of BOP relies on the following: if we execute Adam on y instead of w, we can break the natural bond created by Adam between $\mathrm{sgn}(\hat{\varphi })$ and w—as we saw in the previous sections—responsible for the ant-like behavior of the weights and, consequently, the appearance of side spikes in their histograms. These undesired effects disappear when we de-project y using $X^{-1}$ to get back to the weight vector w:

$$\begin{array}{ccc} w & = & X^{-1}y \end{array}$$

In order to reduce the computational complexity and the memory requirements of this method—recall that *N* is generally a very large number and we must project and de-project the weights on each iteration—we consider X to be a block diagonal matrix with *B* identical $\frac{N}{B}\times \frac{N}{B}$ blocks. In this way, we only have to build and work with a single block $X_{B}$, for which we can choose the size by simply adjusting the value of *B*. The values of this block are drawn from a standard normal distribution. In addition, $X_{B}$ is built as an orthonormal matrix so that $X_{B}^{-1}=X_{B}^{T}$. Let $y^{\left(i\right)}$ and $w^{\left(i\right)}$ be the *i*th block of y and w, respectively, both of them of length $\frac{N}{B}$; therefore, we just compute:

$$\begin{array}{ccc} y^{\left(i\right)} & = & X_{B}w^{\left(i\right)}\\ \nabla_{y^{\left(i\right)}}f\left(y^{\left(i\right)}\right) & = & X_{B}\nabla_{w^{\left(i\right)}}f\left(w^{\left(i\right)}\right) \end{array}$$

After executing Adam, we can get back to $w^{\left(i\right)}$:

$$\begin{array}{ccc} w^{\left(i\right)} & = & X_{B}^{T}y^{\left(i\right)} \end{array}$$

As we will see in Section 6.2.4, BOP does not significantly alter the original distribution of weights, as opposed to standard Adam. This makes it possible to enjoy the advantages of Adam optimization when we implement the watermarking algorithm in [5,6] with a minimal increase in the detectability of the watermark. In addition, this has an advantage in terms of robustness: if the adversary is not able to infer which layer is watermarked, then he/she will have to exert his/her attack (e.g., noise addition, weight pruning) on *every* layer thus producing a larger impact on the performance of the network as measured by the original cost function. We will discuss this fact in the experimental section.

## 5. Information-Theoretic Measures

As already discussed, one of the potential weaknesses of any neural network watermarking algorithm is the detectability of the watermark. An adversary that detects the presence of a watermark on a certain subset of the weights can initiate an attack to remove or alter the watermark. For this reason, it is important that the weights statistically suffer the least modification possible while of course being able to convey the desired hidden message. To measure this statistical closeness, we propose using the KLD [13] between the distributions of weights before and after the watermark embedding. Let *P* and *Q* be two discrete probability distributions defined on the same alphabet X; then, the KLD from *Q* to *P* is (notice that it is not symmetric):

$$\mathrm{KLD}\left(P||Q\right)≐\sum_{x\in X}P\left(x\right)\log\left(\frac{P(x)}{Q(x)}\right)$$

The KLD is always non-negative. The more similar the distributions *P* and *Q* are, the smaller the divergence. In the extreme case of two identical distributions, the divergence is zero.

It is interesting to note that the KLD has been proposed for similar problems in forensics, including steganographic security [23], distinguishability between forensic operators [24], or more general source identification problems [25].

In our case, the two compared distributions are those of $w^{\left(0\right)}$ and $w^{\left(k\right)}$, for *k* just producing convergence with no decoding errors. Since the KLD is not symmetric, it remains to assign those distributions to *P* and *Q* so that the measure is as informative as possible. In particular, we are interested in properly accounting for the possible lateral spikes in the pdf of $w^{\left(k\right)}$. As those spikes often appear where the pdf of $w^{\left(0\right)}$ is small if not negligible, this suggests assigning the latter pdf to *Q* and the former to *P*. However, this choice creates a problem in practice, as for some x∈X, the empirical probabilities are such that P(x)≠0 and Q(x)=0, potentially leading to an infinite divergence. To circumvent this issue related to insufficient sampling, we use an analytical approximation to *Q* with infinite support, after noticing that the empirical distribution of $w^{\left(0\right)}$ with 1000 discrete bins (see Figure 2a) can be approximated by a zero-mean Generalized Gaussian Distribution (GGD) with shape parameter β=0.64 and scale parameter α=0.01, (for notational coherence with the literature, α is used in this section to denote a different quantity than in the rest of the paper.) for which the latter controls the spread of the distribution. As a reference, the KLD between the empirical distribution of $w^{\left(0\right)}$ and its GGD best-fit is 0.0177, which is smaller than any of the KLDs that we find in Table 2. In order to compute the KLD in our experiments, we use this infinite-support symmetric distribution for *Q* and the empirical one of $w^{\left(k\right)}$ for *P* after quantizing both to 1000 discrete bins.

The use of the KLD is adequate to measure the detectability in those cases where the adversary has access to information about the ’expected’ distribution of the weights. For instance, when only one layer is modified, the expected distribution can be inferred from the weights of other layers. However, this may be still too optimistic in terms of adversarial success, as while the expected shape may be preserved—and thus, inferred—across layers, the scale (directly affecting the variance) may be not so. For instance, if the original weights were expected to be zero-mean Gaussian and they still are after watermarking, the KLD (which depends on the ratio of the respective variances) may be quite large, but the adversary will not be able to determine if watermarking took place if he/she does not know what the variance should be and only measures divergence with respect to a Gaussian. To reflect this uncertainty, quite realistic in practical situations, we minimize the KLD with respect to the scale parameter α. This puts the adversary in a scenario where only the shape is used for detectability. Thus, let $Q_{\alpha }$ correspond to a GGD with scale parameter α, then we define the Scale Invariant KLD (SIKLD) as:

$$\mathrm{SIKLD}≐\min_{\alpha }\mathrm{KLD}(P||Q_{\alpha })=\min_{\alpha }\sum_{x\in X}P\left(x\right)\log\left(\frac{P(x)}{Q_{\alpha }\left(x\right)}\right)$$

## 6. Experiments and Results

In this section, we show the experimental results and we compare them to the theory that we have developed. We use MATLAB R2018b to implement the expressions obtained in Section 3 and represent the theoretical histograms. As we will see, both theory and experiments match reasonably well. In particular, for Adam optimization, we are able to reproduce the same position of the side spikes seen in the empirical histograms of Δw, as well as some effects which are attributable to the influence of the denoising cost function. We will also verify the BOP method proposed in Section 4. In addition, the KLD will be computed to give a more precise measure of the similarity between the distributions of $w^{\left(k\right)}$ (i.e., after the embedding) and $w^{\left(0\right)}$, when using SGD, Adam and BOP.

### 6.1. Experimental Set-Up

We employ the fine-tune-to-embed approach described in [5,6]. This means that the training process is divided into two phases, as we explained earlier: (1) training the host network from scratch, and (2) fine-tuning steps for embedding the watermark.

#### 6.1.1. Training the Host Network

In order to perform the initial training of the host network FFDNet, we use the open-source implementation for PyTorch provided in [26]. We employ the FFDNet architecture for color images, which has a depth of 12 convolutional layers and 96 feature maps per layer. The training details are the same as in [26] and also the used datasets: Waterloo Exploration Database [27] for training and Kodak24 [28] for validation. We implement the cost function introduced in (1) and train 80 epochs with the milestones described in [26] on a GPU NVIDIA Titan Xp. We use Adam as the optimization algorithm with its hyperparameters set to their default values. After training the network, we test it on the CBSD68 [29] and Kodak24 datasets.

#### 6.1.2. Watermark Embedding

Once we have trained and tested our host network, we embed our *T*-bit watermark, b=1, T=256, into the convolutional layer l=2 of FFDNet. In the next section, we present the results for both SGD and Adam optimization algorithms. The size of the convolutional filter is 3×3 and the depth of input is I=96, as well as the number of filters in the layer, F=96. Therefore, we have: M=96·3·3=864, and N=MF = 82,944. In addition, the learning rate μ is set to $10^{-6}$ during these fine-tuning steps and, also, we do not perform weight orthogonalization as we did during the initial training.

In addition, we use the following values for the regularizer parameter. When we use SGD we set λ=5 and λ=20 for Gaussian and orthogonal projectors, respectively. In addition, for Adam optimization, we use different values of λ for each configuration to better reflect the influence of the denoising function. In particular, we set λ=0.05 and λ=1 when we use Gaussian projectors, and λ=0.5 and λ=10 when we employ orthogonal projectors. We finish our embedding process when we reach a BER of zero, that is, when all the projected weights are positive—recall that all the embedded bits are set to +1—, i.e., ${\left({\hat{\phi }}^{\left(m\right)}\right)}^{T}w>0$, for all m=1,⋯,T. Notice that these values of λ were selected with the goal of reaching a BER of zero in a relatively fast way and, as it can be seen, they are not straightforwardly comparable for Gaussian and orthogonal projectors. Finally, to check the validity of our proposed method BOP, we use the same values of λ as with Adam optimization, and set the number of blocks *B* to 12.

### 6.2. Experimental Results

Here, we present the experimental results. After the main training of the host network and before the watermark embedding, we obtain a PSNR of 31.18 dB and 32.13 dB on the CBSD68 and Kodak24 datasets, respectively, for a noise level of 25. These results are very close to those reported in [26]. Compare these values to those in Table 2, where we show the PSNR (dB) results on the CBSD68 and Kodak24 datasets for the same noise level after the watermark embedding was performed. As we see, when we embed the watermark using SGD with Gaussian projectors, the denoising performance drops about 0.45 dB, while, if we employ orthogonal projectors, the performance drops only 0.1 dB. Thus, employing orthogonal projectors with SGD optimization might be beneficial to better preserve the denoising performance. For Adam optimization and BOP, the original performance does not significantly drop and it even increases when using orthogonal projectors. Consequently, in order to keep a good performance after the watermark embedding, Adam would be preferable to SGD were it not for the conspicuousness of the weights. Our proposed method BOP is a good solution to bring the detectability of Adam down to similar levels as SGD and still enjoy the rest of advantages.

Table 2 also presents the number of iterations required for obtaining a BER of zero and the KLD and Scale Invariant KLD (SIKLD) between the distributions of $w^{\left(k\right)}$ and $w^{\left(0\right)}$ for each configuration.

#### 6.2.1. Empirical Denoising Gradients

**Figure 3:**
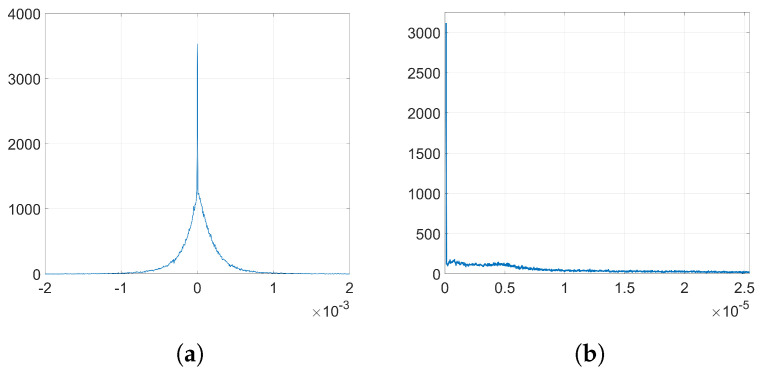
Empirical histograms from the denoising gradients (**a**) distribution of the mean denoising gradient, *D*; (**b**) distribution of the variance of the batching noise, *H*.

In order to analyze the influence of the denoising function, we need to get the empirical distributions of the mean denoising gradient and the variance of the batching noise. To that end, we proceed as follows: firstly, we extract the denoising gradients from the embedding layer l=2 and we average them for each coefficient over the number of iterations *k* to get the distribution of the mean. Then, the batching noise can be easily computed if, for each coefficient, we subtract the mean value from its corresponding denoising gradient value at each iteration. By computing the variance of this noise for each individual weight, we can estimate the overall distribution of the variance of the batching noise. Figure 3 shows the empirical distribution of the mean denoising gradient, *D*, and the variance of the batching noise, *H*.

#### 6.2.2. SGD

**Figure 4:**
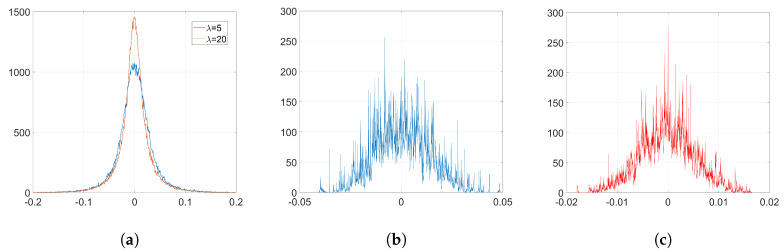
Empirical histograms after the watermark embedding using SGD. (**a**) histogram of $w^{\left(k\right)}$, Gaussian, λ=5, and orthogonal projectors, λ=20; (**b**) histogram of Δw, Gaussian, λ=5; (**c**) histogram of Δw, orthogonal, λ=20.

We embed our watermark using SGD and its corresponding set-up, as we detailed in Section 6.1.2. We show the resulting histograms of $w^{\left(k\right)}$ and Δw in Figure 4. As we can see, SGD does not significantly alter the original distribution of weights shown in Figure 2a. This is also reflected in the SIKLD, which is very small, especially when we use orthogonal projectors (see Table 2).

**Figure 5:**
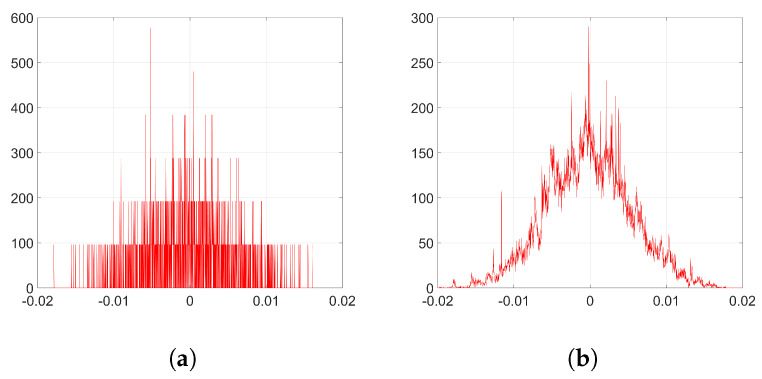
Theoretical histograms of Δw for SGD, orthogonal projectors, λ=20. (**a**) only watermarking function, Equation (24); (**b**) including denoising and watermarking functions, Equation (48).

Now, we check the theory we developed for SGD in Section 3.1. Recall that out theoretical analysis just covers the case of orthogonal projectors. Using (24), we can generate samples of Δw without including the effect of the denoising cost function. The resulting histogram is shown in Figure 5a. Notice that the unusual appearance of this histogram can be attributed to the effects of applying the initial transformation explained in Section 2.3.1. In particular, each value of φ repeats *F* times to form vector $\hat{\varphi }$; hence, the discrete values in the *y*-axis of the histogram shown in Figure 5a. However, we see that the range of values of this theoretical histogram fits quite well the empirical one (Figure 4c). In order to get a more accurate representation, we can generate samples of Δw according to (48), so that we add the effect of noise coming from the denoising cost function. As we see in Figure 5b, the resulting histogram is now very similar to the one in Figure 4c.

In addition, we confirm that (25) can be used to compute the variance of the distribution of $w^{\left(k\right)}$ when we implement orthogonal projectors. Firstly, we check the hypothesis that we made regarding the uncorrelatedness between $w^{\left(0\right)}$ and Δw. For our particular case of λ=20, the correlation coefficient between $w^{\left(0\right)}$ and Δw is $2.539\cdot 10^{-4}$, a very small value that confirms our assumption. Using (25), we have that the variance of the empirical distribution of $w^{\left(k\right)}$—red histogram in Figure 4c—is $1.192\cdot 10^{-3}$ while the theoretical variance is $1.193\cdot 10^{-3}$. As we see, these values are almost identical.

#### 6.2.3. Adam

**Figure 6:**
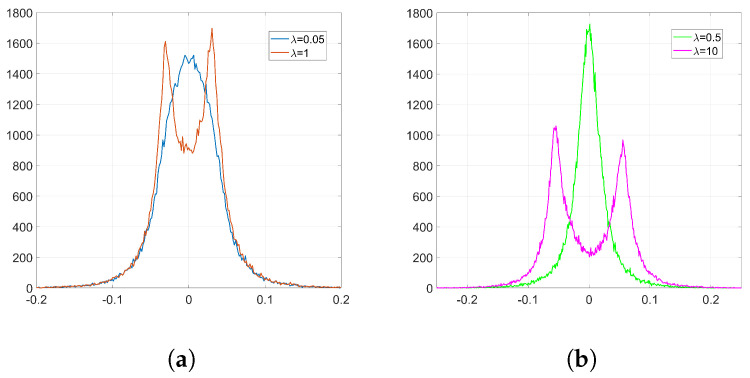
Empirical histograms of $w^{\left(k\right)}$ after the watermark embedding using Adam. (**a**) Gaussian, λ=0.05 and λ=1; (**b**) orthogonal, λ=0.5 and λ=10.

**Figure 7:**
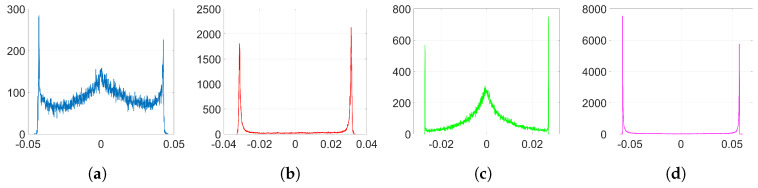
Empirical histograms of Δw after the watermark embedding using Adam. (**a**) Gaussian, λ=0.05; (**b**) Gaussian, λ=1; (**c**) orthogonal, λ=0.5; (**d**) orthogonal, λ=10.

In the following experiments, we employ Adam optimization for the watermark embedding and use the same settings as in Section 6.1.2. The resulting histograms of $w^{\left(k\right)}$ and Δw are shown in Figure 6 and Figure 7, respectively. As it can be observed, the shape of the weight distribution changes to a great extent for λ=1 and λ=10. For smaller values of λ, since the influence of the watermarking cost function is weaker, we can avoid having a significant alteration to the original distribution shape. This is also reflected on their SIKLD values in Table 2: as λ increases the SIKLD also increases considerably. However, notice that, whatever the value of λ is, the histograms of weight variations always present the characteristic side spikes. These footprints left by Adam can increase the detectability of the watermark. In addition, when λ is small, we can observe in these histograms the influence of the denoising cost function: it causes the appearance of a central peak with values that spread till the location of both side spikes.

**Figure 8:**
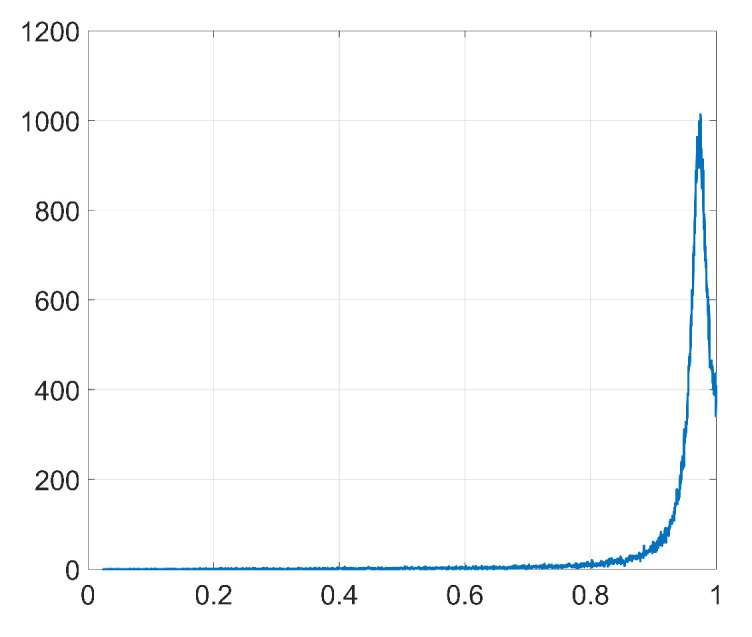
Pdf of Γ, Gaussian projectors.

**Figure 9:**
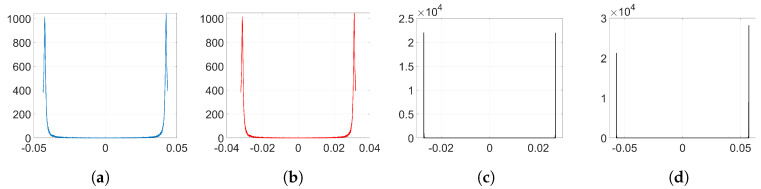
Theoretical histograms of Δw for Adam with only watermarking function using Equations (43) and (44). (**a**) Gaussian, λ=0.05; (**b**) Gaussian, λ=1; (**c**) orthogonal, λ=0.5; (**d**) orthogonal, λ=10.

Figure 8 represents the pdf of Γ obtained from (43). Notice that, as we stated in Section 3.2.5, the pdf is concentrated around its mode. Figure 9 shows the histograms of Δw obtained from (44) when only the watermarking loss $\tilde{f}\left(w\right)$ is optimized and the denoising component is set to zero. Compare these histograms to those in Figure 7: the theory developed in Section 3.2 is able to explain the two-spiked distributions of Δw. Notice that these theoretical expressions provide a good enough approximation since they allow us to predict the position of the side spikes. We show in Table 3 the values of these positions obtained from both theoretical (Figure 9) and empirical (Figure 7) results. As we can see, these side spikes are placed in almost identical positions by both theory and experiments, hence, we can confirm that the sign phenomenon in Adam is responsible for this ant-like behavior shown by the weights at the embedding layer.

**Figure 10:**
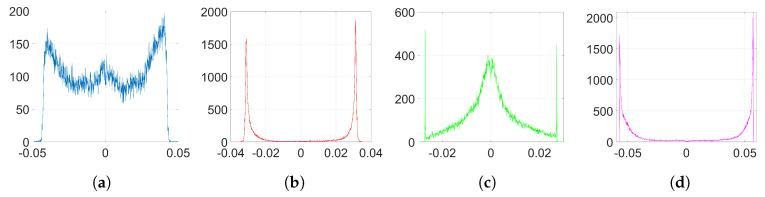
Theoretical histograms of Δw for Adam with denoising and watermarking functions using Equations (A11) and (A12). (**a**) Gaussian, λ=0.05; (**b**) Gaussian, λ=1; (**c**) orthogonal, λ=0.5; (**d**) orthogonal, λ=10.

Finally, in order to reflect the influence of the denoising function and obtain more realistic histograms, we can solve the fourth-degree Equation (A11) and, then, generate samples of Δw according to (A12). The resulting histograms are shown in Figure 10 and are now quite similar to those in Figure 7. As it can be seen, we can emulate the central peak and the dispersion of the values of the side spikes in the histograms and, thus, we can confirm that these effects are attributable to the influence of the denoising cost function.

#### 6.2.4. BOP

**Figure 11:**
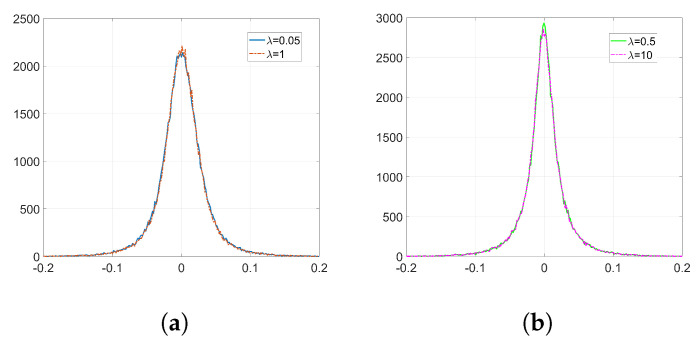
Empirical histograms of $w^{\left(k\right)}$ after the watermark embedding using Adam. (**a**) Gaussian, λ=0.05 and λ=1; (**b**) orthogonal, λ=0.5 and λ=10.

**Figure 12:**
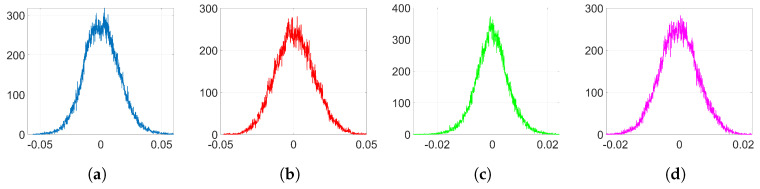
Empirical histograms of Δw after the watermark embedding using Adam. (**a**) Gaussian, λ=0.05; (**b**) Gaussian, λ=1; (**c**) orthogonal, λ=0.5; (**d**) orthogonal, λ=10.

Here, we represent the empirical histograms when we implement our method BOP. Figure 11 and Figure 12 show the histograms of $w^{\left(k\right)}$ and Δw, respectively. As we see from these histograms and the KLD and SIKLD values in Table 2, this method allows us to remove the side spikes of the histograms and much better preserve the original shape of the weight distribution. As a result, the detectability of the watermark due to Adam optimization is strongly reduced.

**Figure 13:**
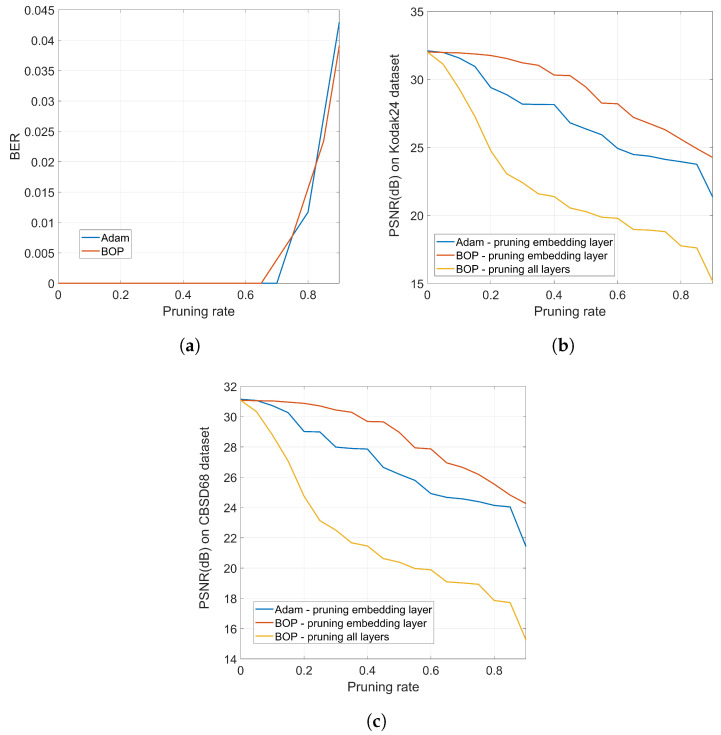
(**a**) BER vs. pruning rate for Adam and BOP (pruning all layers or only the watermarked one does not have any impact on BER); (**b**) PSNR vs. pruning rate for Adam and BOP for the Kodak24 dataset; (**c**) PSNR vs. pruning rate for Adam and BOP for the CBSD68 dataset.

A positive side effect of the undetectability of the watermark is that the robustness is increased because an adversary will not know which layer must be modified in order to alter the embedded watermark. This is illustrated in Figure 13 where we compare the robustness of standard Adam with that of BOP against weight pruning. The network is trained long enough for both Adam and BOP to guarantee a similar BER vs. pruning rate, as shown in Figure 13a. Then, the PSNR obtained after training and pruning is shown in Figure 13b for the Kodak24 dataset which illustrates the following facts: (1) BOP produces a network that is more robust to pruning in terms of PSNR, which can be valuable towards model compression; for instance, for a pruning rate of 0.35 (that has no impact on the BER of the hidden information), BOP degrades the original PSNR by about 1 dB, whereas Adam would produce a degradation of more than 3 dB. (2) This robustness might be detrimental in case it is an attacker who does the pruning in an attempt to degrade the watermark; for instance, for a pruning rate of 0.82, the BER for both Adam and BOP rises to 0.02 (see Figure 13a). For this pruning rate, the PSNR of BOP is around 25 dB while Adam gives slightly less than 24 dB. Then, in the case of BOP, the adversary would be able to produce a network that performs closer to the original in terms of PSNR—notice, however, that the degradation in both cases is quite severe, so this heavy pruning would render a denoiser with little practical use —. (3) In any case, the previous comparison would assume that the adversary knows the layer that contains the watermark; as we have properly justified, this is reasonable for Adam but not so for BOP. If the adversary does not know the layer that must be pruned, then, to achieve the same target BER, he/she must prune all the layers. In this case, for the same pruning rate of 0.82 that causes the BER to increase to 0.02, the PSNR drops to less than 18 dB.

Similar conclusions can be extracted from Figure 13c that shows the PSNR vs. pruning rate for the CBSD68 dataset. These experiments clearly show the higher robustness brought about by the undetectability of BOP, as it prevents attacks targeted to a specific layer.

## 7. Conclusions

Throughout this paper, we have shown the importance of being careful with the optimization algorithm when we embed watermarks following the approach in [5,6]. The choice of certain optimization algorithms whose update direction is given by the sign function can originate footprints in the distributions of weights that are easily detectable by adversaries, thus compromising the efficacy of the watermarking algorithm.

In particular, we studied the mechanisms behind SGD and Adam optimization and found that the sign phenomenon that occurs in Adam is detrimental for watermarking, since it causes the appearance of two salient side spikes on the histograms of weights. As opposed to Adam, the sign function does not appear when we use SGD. Therefore, SGD does not significantly alter the original shape of the distribution of weights although, as we showed in the theoretical analysis, it slightly increases its variance. The analysis in this paper can be extended to other optimization algorithms.

In addition, we introduced orthogonal projectors and observed that, compared to the Gaussian case, they generally preserve the original performance and weight distribution better. However, a deeper analysis on this subject is left for further research.

Finally, we presented a novel method that uses orthogonal block projections to address the use of Adam optimization together with the watermarking algorithm under study. As we checked in the empirical section, this method allows us to solve the detectability problem posed by Adam and still enjoy the rest of advantages of this optimization algorithm.

## Figures and Tables

**Table 1:** FFDNet configurations for grayscale and RGB image denoising.

	Grayscale	RGB
Conv layers	15	12
Feature maps per layer	64	96
Receptive field	62×62	50×50

**Table 2:** PSNR (dB) results with noise level σ=25, number of iterations *k* needed to converge, KLD and SIKLD between the distributions of $w^{\left(k\right)}$ and $w^{\left(0\right)}$.

	SGD		Adam				BOP			
	Gaussian	Orth.	Gaussian		Orth.		Gaussian		Orth.	
λ	5	20	0.05	1	0.5	10	0.05	1	0.5	10
CBSD68	30.76	31.09	31.18	31.17	31.21	31.16	31.20	31.16	31.19	31.15
Kodak24	31.66	32.03	32.13	32.10	32.15	32.10	32.15	32.08	32.14	32.09
*k*	42,780	98,510	43,590	32,140	27,110	57,230	40,880	14,840	33,180	7150
KLD	0.0477	0.0238	0.1149	0.2779	0.0281	0.8118	0.0463	0.0443	0.0227	0.0253
SIKLD	0.0468	0.0206	0.0879	0.2112	0.0280	0.4707	0.0449	0.0266	0.0197	0.0226

**Table 3:** Position of both side spikes in the histograms of Δw obtained from theoretical and empirical results.

	λ	Theoretical	Empirical
**Gaussian**	0.05	−0.04243	−0.04278
		0.04239	0.04276
	1	−0.03129	−0.03119
		0.03126	0.03125
**Orthogonal**	0.5	−0.02710	−0.02710
		0.02710	0.02709
	10	−0.05720	−0.05717
		0.05720	0.05718

**Figure A1:**
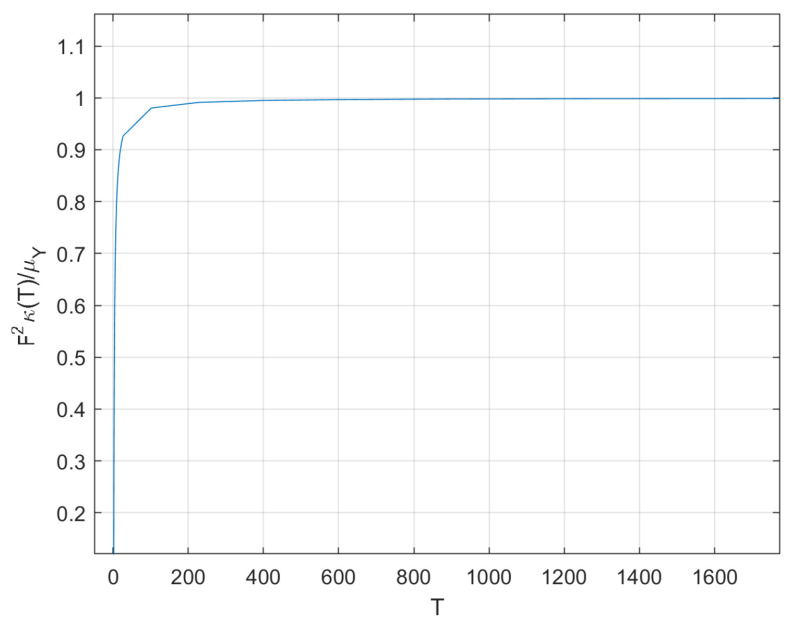
$F^{2}\cdot \kappa \left(T\right)/\mu_{Y}$ vs. *T*

**Figure A2:**
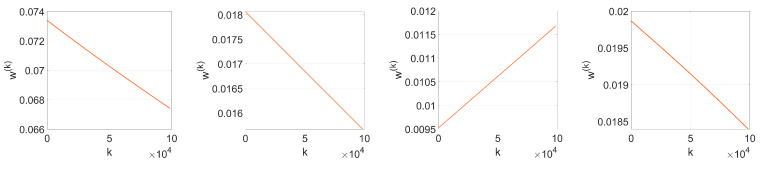
Evolution with *k* of four randomly selected weights. SGD optimization with orthogonal projectors, λ=20, T=256.

**Figure A3:**
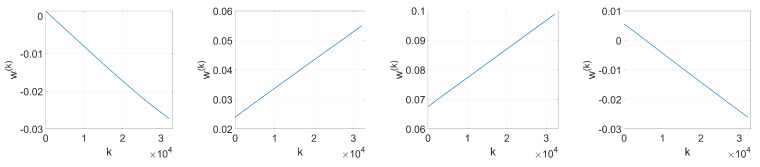
Evolution with *k* of four randomly selected weights. Adam optimization with Gaussian projectors, λ=1, T=256.

**Figure A4:**
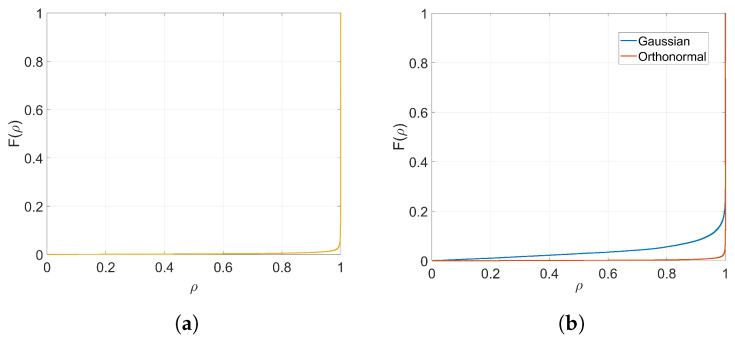
ECDF of the correlation coefficient ρ between the observed values of the weights over *k* and their predicted affine evolution. (**a**) SGD with orthogonal projectors, λ=20; (**b**) Adam with Gaussian, λ=1 and orthogonal projectors, λ=10.

## Appendix A. Mathematical Derivations

### Appendix A.1. Projected Weights at k = *0*

We want to compute the mean and the variance of ${\hat{\Phi }}^{T}w^{\left(0\right)}$. It is easy to see that the mean is zero for any ${\left({\hat{\phi }}^{\left(i\right)}\right)}^{T}w^{\left(0\right)}$, i=1,⋯,T because $E\{{\hat{\phi }}_{j}^{\left(i\right)}\}=0$.

Now, in order to obtain an expression for the variance, we may compute the expectation of the trace of the covariance matrix of ${\hat{\Phi }}^{T}w^{\left(0\right)}$ divided by *T*. Therefore, if we use the properties of the trace, we have:

$$\begin{array}{ccc} \mathrm{Var}\{{\hat{\Phi }}^{T}w^{\left(0\right)}\} & = & \frac{1}{T}E\left\{\mathrm{Tr}\left[{\hat{\Phi }}^{T}w^{\left(0\right)}{\left(w^{\left(0\right)}\right)}^{T}\hat{\Phi }\right]\right\}\\ & = & \frac{1}{T}E\left\{\mathrm{Tr}\left[w^{\left(0\right)}{\left(w^{\left(0\right)}\right)}^{T}\hat{\Phi }{\hat{\Phi }}^{T}\right]\right\}\\ & = & \frac{1}{T}\sum_{i,j}E\left\{{\left(w^{\left(0\right)}{\left(w^{\left(0\right)}\right)}^{T}\right)}_{i,j}{\left(\hat{\Phi }{\hat{\Phi }}^{T}\right)}_{i,j}\right\} \end{array}$$

We can write:

(A1) $$E\left\{{\left(w^{\left(0\right)}{\left(w^{\left(0\right)}\right)}^{T}\right)}_{i,j}{\left(\hat{\Phi }{\hat{\Phi }}^{T}\right)}_{i,j}\right\}=E\left\{w_{i}^{\left(0\right)}w_{j}^{\left(0\right)}\right\}E\left\{\sum_{m=1}^{T}{\hat{\phi }}_{i}^{\left(m\right)}{\hat{\phi }}_{j}^{\left(m\right)}\right\}$$

It is straightforward to see that, for the off-diagonal terms, i.e., i≠j, the expectation in (A1) is zero. For the *N* diagonal terms, i.e., i=j, we have instead:

$$E\left\{{\left(w_{i}^{\left(0\right)}\right)}^{2}\right\}E\left\{\sum_{m=1}^{T}{\left({\hat{\phi }}_{i}^{\left(m\right)}\right)}^{2}\right\}=T\cdot \mathrm{Var}\left\{w^{\left(0\right)}\right\}\mathrm{Var}\left\{{\hat{\phi }}^{\left(m\right)}\right\}$$

where $\mathrm{Var}\{{\hat{\phi }}^{\left(m\right)}\}$ is the variance of any projection vector ${\hat{\phi }}^{\left(m\right)}$, m=1,⋯,T. When we use Gaussian projectors, we have $\mathrm{Var}\left\{{\hat{\phi }}^{\left(m\right)}\right\}=1/F^{2}$ and when we use orthogonal projectors, we have $\mathrm{Var}\left\{{\hat{\phi }}^{\left(m\right)}\right\}\;=\;1/\left(FN\right)$. Therefore, the variance for Gaussian projectors is

$$\mathrm{Var}\left\{{\hat{\Phi }}^{T}w^{\left(0\right)}\right\}=\frac{N}{F^{2}}\mathrm{Var}\left\{w^{\left(0\right)}\right\}$$

while for orthogonal projector is

$$\mathrm{Var}\left\{{\hat{\Phi }}^{T}w^{\left(0\right)}\right\}=\frac{1}{F}\mathrm{Var}\left\{w^{\left(0\right)}\right\}$$

### Appendix A.2. Adam: Mean of the Gradient

In order to find an expression for the mean of the gradient, we substitute (17) into (26) and obtain:

$$\begin{array}{ccc} m^{\left(k\right)} & = & -\frac{\lambda }{2}\cdot \hat{\varphi }\cdot \left(1-\beta_{1}\right)\sum_{i=1}^{k}\beta_{1}^{k-i}+\frac{\lambda }{4}\cdot \hat{\Psi }\cdot \left(1-\beta_{1}\right)\sum_{i=1}^{k}\beta_{1}^{k-i}w^{\left(i\right)}\\ & = & -\frac{\lambda }{2}\cdot \hat{\varphi }\cdot \left(1-\beta_{1}^{k}\right)+\frac{\lambda }{4}\cdot \hat{\Psi }\cdot \left(1-\beta_{1}\right)\sum_{i=1}^{k}\beta_{1}^{k-i}w^{\left(i\right)} \end{array}$$

The bias-corrected mean gradient is:

$$\begin{array}{c} {\hat{m}}^{\left(k\right)}=\frac{m^{\left(k\right)}}{1-\beta_{1}^{k}}=-\frac{\lambda }{2}\hat{\varphi }+\frac{\lambda }{4}\cdot \hat{\Psi }\cdot \frac{1-\beta_{1}}{1-\beta_{1}^{k}}\sum_{i=1}^{k}\beta_{1}^{k-i}w^{\left(i\right)} \end{array}$$

The main difficulty in finding an explicit expression for ${\hat{m}}^{\left(k\right)}$ is the last sum which requires a closed-form expression for $w^{\left(i\right)}$, for all i=0,⋯,k, which in turn depends on the Adam adaptation. This easily leads to a difference equation whose solution is cumbersome. Alternatively, we start by conjecturing the affine-growth in (18) and write:

$$\begin{array}{ccc} \frac{1-\beta_{1}}{1-\beta_{1}^{k}}\sum_{i=1}^{k}\beta_{1}^{k-i}w^{\left(i\right)} & = & \frac{1-\beta_{1}}{1-\beta_{1}^{k}}\sum_{i=1}^{k}\beta_{1}^{k-i}\left(w^{\left(0\right)}+i\cdot \mu \eta \right)\\ & = & w^{\left(0\right)}+\mu \eta \frac{1-\beta_{1}}{1-\beta_{1}^{k}}\sum_{i=1}^{k}i\cdot \beta_{1}^{k-i}\\ & = & w^{\left(0\right)}+\mu \eta \left(\frac{k}{1-\beta_{1}^{k}}-\frac{\beta_{1}}{1-\beta_{1}}\right) \end{array}$$

Therefore, under the affine-growth hypothesis, we can write:

$$\begin{array}{c} {\hat{m}}^{\left(k\right)}=-\frac{\lambda }{2}\hat{\varphi }+\frac{\lambda }{4}\cdot \hat{\Psi }\left(w^{\left(0\right)}+\mu \eta \left(\frac{k}{1-\beta_{1}^{k}}-\frac{\beta_{1}}{1-\beta_{1}}\right)\right) \end{array}$$

and arrive at the expression in (27).

### Appendix A.3. Adam: Variance of the Gradient

To get an expression for the variance of the gradient, we plug (31) into (34) and write:

$$\begin{array}{ccc} v_{j}^{\left(k\right)} & = & \frac{\lambda^{2}}{4}\left(1-\beta_{2}\right)\sum_{i=1}^{k}\beta_{2}^{k-i}\left(p_{j}+q_{j}\mu i+r_{j}\mu^{2}i^{2}\right)\\ & = & \frac{\lambda^{2}}{4}\left(1-\beta_{2}\right)p_{j}\sum_{i=1}^{k}\beta_{2}^{k-i}+\frac{\lambda^{2}}{4}\left(1-\beta_{2}\right)q_{j}\mu \sum_{i=1}^{k}i\beta_{2}^{k-i}+\frac{\lambda^{2}}{4}\left(1-\beta_{2}\right)r_{j}\mu^{2}\sum_{i=1}^{k}i^{2}\beta_{2}^{k-i}\\ & = & \frac{\lambda^{2}}{4}\left(1-\beta_{2}^{k}\right)\left(p_{j}-\frac{\beta_{2}}{1-\beta_{2}}\mu q_{j}+\frac{\beta_{2}+\beta_{2}^{2}}{{\left(1-\beta_{2}\right)}^{2}}\mu^{2}r_{j}\right)+\frac{\lambda^{2}}{4}k\left(\mu q_{j}-\frac{2\beta_{2}}{1-\beta_{2}}\mu^{2}r_{j}\right)+\frac{\lambda^{2}}{4}k^{2}\mu^{2}r_{j} \end{array}$$

### Appendix A.4. Adam: A Projection-Based Decomposition of $c_{j}^{T}\mathrm{sgn}\left(\hat{\varphi }\right)$

We want to write $c_{j}^{T}\mathrm{sgn}\left(\hat{\varphi }\right)=\alpha \cdot {\hat{\varphi }}_{j}+z_{j}$. To find α, we compute the cross-correlation with $\hat{\varphi }$, i.e., $E\left\{{\hat{\varphi }}^{T}C^{T}\mathrm{sgn}\left(\hat{\varphi }\right)\right\}=\alpha E\{||\hat{\varphi }{\left|\right|}^{2}\}$. Then, z is simply $C^{T}\mathrm{sgn}\left(\hat{\varphi }\right)-\alpha \cdot \hat{\varphi }$. In the following, we show separately the derivations for the Gaussian and orthogonal projectors.

#### Appendix A.4.1. Decomposition for Gaussian Projectors

Recall that the projection vectors have i.i.d. components drawn from a N(0,1) distribution. Since the quantity of interest $E\{{\hat{\varphi }}^{T}C^{T}\mathrm{sgn}\left(\hat{\varphi }\right)\}$ is a scalar, we will use the trace to manipulate the involved matrices, as follows:

$$\begin{array}{ccc} E\{{\hat{\varphi }}^{T}C^{T}\mathrm{sgn}\left(\hat{\varphi }\right)\} & = & \mathrm{Tr}\left[E\{{\hat{\varphi }}^{T}C^{T}\mathrm{sgn}\left(\hat{\varphi }\right)\}\right]=E\left\{\mathrm{Tr}[C^{T}\mathrm{sgn}\left(\hat{\varphi }\right){\hat{\varphi }}^{T}]\right\}\\ & = & \sum_{i,j}E\left\{{\left(C\circ (\mathrm{sgn}\left(\hat{\varphi }\right){\hat{\varphi }}^{T})\right)}_{i,j}\right\}=\sum_{i,j}\sum_{m=1}^{T}\sum_{n=1}^{T}E\left\{{\hat{\phi }}_{i}^{\left(m\right)}\mathrm{sgn}\left({\hat{\varphi }}_{i}\right){\hat{\phi }}_{j}^{\left(m\right)}{\hat{\phi }}_{j}^{\left(n\right)}\right\} \end{array}$$

where ${\hat{\phi }}_{i}^{\left(m\right)}=\theta_{i}\phi^{\left(m\right)}$, m=1,⋯,T, i=1,⋯,N, and $\theta_{i}$ is the *i*th row of matrix Θ. In order to compute the previous expectation, we need to consider separately the diagonal terms and the off-diagonal ones.

We start with the off-diagonal elements (i.e., i≠j). Here, we also need to distinguish the following cases: $\left\lfloor \frac{i}{F}\right\rfloor \neq \left\lfloor \frac{j}{F}\right\rfloor$, satisfied by N(N−F) elements, and $\left\lfloor \frac{i}{F}\right\rfloor =\left\lfloor \frac{j}{F}\right\rfloor$, which applies for the remaining N(F−1) off-diagonal terms. Therefore, in the first subcase, we have that $\theta_{i}\neq \theta_{j}$, so ${\hat{\phi }}_{i}^{\left(m\right)}\neq {\hat{\phi }}_{j}^{\left(m\right)}$ and:

(A2) $$\sum_{m=1}^{T}\sum_{n=1}^{T}E\left\{{\hat{\phi }}_{i}^{\left(m\right)}\mathrm{sgn}\left({\hat{\varphi }}_{i}\right){\hat{\phi }}_{j}^{\left(m\right)}{\hat{\phi }}_{j}^{\left(n\right)}\right\}=\sum_{m=1}^{T}E\left\{{\hat{\phi }}_{i}^{\left(m\right)}\mathrm{sgn}\left({\hat{\varphi }}_{i}\right)\right\}E\left\{{\left({\hat{\phi }}_{j}^{\left(m\right)}\right)}^{2}\right\}$$

In order to compute the expectation $E\{{\hat{\phi }}_{i}^{\left(m\right)}\mathrm{sgn}\left({\hat{\varphi }}_{i}\right)\}$ in (A2), we write:

$$\begin{array}{ccc} {\hat{\varphi }}_{i} & = & {\hat{\phi }}_{i}^{\left(m\right)}+\sum_{\overset{l=1}{i\neq m}}^{T}{\hat{\phi }}_{i}^{\left(l\right)}\\ & = & {\hat{\phi }}_{i}^{\left(m\right)}+n_{i} \end{array}$$

where $n_{i}$ can be seen as a realization of an i.i.d. Gaussian distribution with zero-mean and variance $\left(T-1\right)/F^{2}$. Assume without loss of generality that ${\hat{\phi }}_{i}^{\left(m\right)}>0$. Then, the probability that $\mathrm{sgn}({\hat{\varphi }}_{i})=-1$ is $p≐Q\left({\hat{\phi }}_{i}^{\left(m\right)}F/\sqrt{T-1}\right)$, ($Q\left(x\right)≐\frac{1}{\sqrt{2\pi }}\int_{x}^{\infty }e^{-x^{2}/2}dx$.) and ${\hat{\phi }}_{i}^{\left(m\right)}\mathrm{sgn}\left({\hat{\varphi }}_{i}\right)$ will take the value $-{\hat{\phi }}_{i}^{\left(m\right)}$ with probability *p*, and ${\hat{\phi }}_{i}^{\left(m\right)}$ with probability 1−p. Then, the distribution of ${\hat{\phi }}_{i}^{\left(m\right)}\mathrm{sgn}\left({\hat{\varphi }}_{i}\right)$ will correspond to that of a random variable *Y* constructed as follows: letting $X∼N(0,1/F^{2})$, we define *Y* as $Y\;≐\;|X|\cdot \left(1-2Q(|X|F/\sqrt{T-1})\right)$. Then,

$$E\left\{Y\right\}=\frac{F}{\sqrt{2\pi }}\int_{0}^{\infty }2x\left(1-2Q\left(\frac{xF}{\sqrt{T-1}}\right)\right)e^{-x^{2}F^{2}/2}dx$$

It can be shown that this integral gives [30]:

(A3) $$E\left\{Y\right\}=\frac{1}{F}\sqrt{\frac{2}{\pi T}}≐\mu_{Y}$$

Therefore, when i≠j and $\left\lfloor \frac{i}{F}\right\rfloor \neq \left\lfloor \frac{j}{F}\right\rfloor$, we can compute (A2) as:

$$\sum_{m=1}^{T}E\left\{{\hat{\phi }}_{i}^{\left(m\right)}\mathrm{sgn}\left({\hat{\varphi }}_{i}\right)\right\}E\left\{{\left({\hat{\phi }}_{j}^{\left(m\right)}\right)}^{2}\right\}=\frac{T}{F^{2}}\mu_{Y}$$

When i≠j and $\left\lfloor \frac{i}{F}\right\rfloor =\left\lfloor \frac{j}{F}\right\rfloor$, we have that $\theta_{i}=\theta_{j}$, so ${\hat{\phi }}_{i}^{\left(m\right)}={\hat{\phi }}_{j}^{\left(m\right)}$. Thus:

(A4) $$\begin{array}{ccc} \sum_{m=1}^{T}\sum_{n=1}^{T}E\left\{{\left({\hat{\phi }}_{i}^{\left(m\right)}\right)}^{2}{\hat{\phi }}_{i}^{\left(n\right)}\mathrm{sgn}\left({\hat{\varphi }}_{i}\right)\right\} & = & \sum_{m=1}^{T}E\left\{{\left({\hat{\phi }}_{i}^{\left(m\right)}\right)}^{3}\mathrm{sgn}\left({\hat{\varphi }}_{i}\right)\right\}+\sum_{m=1}^{T}\sum_{\overset{n=1}{n\neq m}}^{T}E\left\{{\left({\hat{\phi }}_{i}^{\left(m\right)}\right)}^{2}{\hat{\phi }}_{i}^{\left(n\right)}\mathrm{sgn}\left({\hat{\varphi }}_{i}\right)\right\}\\ & = & T\xi_{Y^{\prime }}+T\left(T-1\right)E\left\{{\left({\hat{\phi }}_{i}^{\left(m\right)}\right)}^{2}{\hat{\phi }}_{i}^{\left(n\right)}\mathrm{sgn}\left({\hat{\varphi }}_{i}\right)\right\} \end{array}$$

where $\xi_{Y^{\prime }}$ can be computed as the mean of $Y^{\prime }$ defined in a similar way as above, i.e.,: let $X∼N(0,1/F^{2})$, we define $Y^{\prime }$ as $Y^{\prime }≐{\left|X\right|}^{3}\cdot \left(1-2Q(|X|F/\sqrt{T-1})\right)$. Then:

$$\xi_{Y^{\prime }}≐E\left\{Y^{\prime }\right\}=\frac{F}{\sqrt{2\pi }}\int_{0}^{\infty }2x^{3}\left(1-2Q\left(\frac{xF}{\sqrt{T-1}}\right)\right)e^{-x^{2}F^{2}/2}dx$$

Using Mathematica, it is found that:

(A5) $$\xi_{Y^{\prime }}=\sqrt{\frac{2}{\pi T}}\frac{3T-1}{TF^{3}}=\frac{\left(3T-1\right)}{TF^{2}}\mu_{Y}$$

Unfortunately, the double integral required to compute the expectation in (A4) does not seem to admit a closed-form solution. On the other hand, its numerical computation through Monte Carlo integration is rather straightforward. Let $X,Y∼N(0,1/F^{2})$ and $Z∼N(0,\frac{T-2}{F^{2}})$, all mutually independent, then the desired expectation is $E\{XY^{2}\mathrm{sgn}\left(X+Y+Z\right)\}≐\kappa \left(T\right)$, where, with κ(T), we stress the fact that the integral depends on *T* alone. The result is represented in Figure A1. It is possible to show that, as T→∞, $\kappa \left(T\right)\to \frac{\mu_{Y}}{F^{2}}$.

Then, for moderately large *T*, the sum in (A4) is approximately $\mu_{Y}\left(T^{2}+2T-1\right)/F^{2}$.

For the i=j case (i.e., diagonal terms), we obtain the same result as (A4). Therefore, when computing $\sum_{i,j}E\left\{{\left(C\circ (\mathrm{sgn}\left(\hat{\varphi }\right){\hat{\varphi }}^{T})\right)}_{i,j}\right\}$, there are N(N−F) terms with value $\mu_{Y}T/F^{2}$, and NF terms with approximate value $\mu_{Y}\left(T^{2}+2T-1\right)/F^{2}$, hence:

$$\begin{array}{c} E\left\{{\hat{\varphi }}^{T}C^{T}\mathrm{sgn}\left(\hat{\varphi }\right)\right\}\approx \frac{N\mu_{Y}}{F^{2}}\left(NT+F\left(T^{2}+T-1\right)\right) \end{array}$$

In addition, $E\{||\hat{\varphi }{\left|\right|}^{2}\}\}=E\left\{{\hat{\varphi }}^{T}\hat{\varphi }\right\}=E\left\{\varphi^{T}\Theta^{T}\Theta \varphi \right\}=\frac{MT}{F}=\frac{NT}{F^{2}}$, so we have:

(A6) $$\begin{array}{ccc} \alpha & = & \frac{E\{{\hat{\varphi }}^{T}C^{T}\mathrm{sgn}\left(\hat{\varphi }\right)\}}{E\{||\hat{\varphi }|{|}^{2}\}\}}\approx \frac{F^{2}N\mu_{Y}\left(NT+F\left(T^{2}+T-1\right)\right)}{F^{2}NT}\\ & = & \frac{\mu_{Y}}{T}\left(FT^{2}+\left(N+F\right)T-F\right) \end{array}$$

We are interested now in measuring the variance of $z_{j}$, j=1,⋯,N, which, by construction, we assume to be i.i.d. First, we note that the covariance matrix is:

$$C^{T}\mathrm{sgn}\left(\hat{\varphi }\right)\mathrm{sgn}\left({\hat{\varphi }}^{T}\right)C-\alpha^{2}\hat{\varphi }{\hat{\varphi }}^{T}$$

Then, the variance of $z_{j}$ will be the expectation of the trace of this matrix divided by *N*. The second term is immediate to compute, as $E\left\{\mathrm{Tr}\left[\hat{\varphi }{\hat{\varphi }}^{T}\right]\right\}=NT/F^{2}$, so we focus next on $\mathrm{Tr}\left[C^{T}\mathrm{sgn}\left(\hat{\varphi }\right)\mathrm{sgn}\left({\hat{\varphi }}^{T}\right)C\right]=\mathrm{Tr}\left[CC^{T}\mathrm{sgn}\left(\hat{\varphi }\right)\mathrm{sgn}\left({\hat{\varphi }}^{T}\right)\right]$. Again, we can write:

$$E\left\{\mathrm{Tr}\left[CC^{T}\mathrm{sgn}\left(\hat{\varphi }\right)\mathrm{sgn}\left({\hat{\varphi }}^{T}\right)\right]\right\}=E\left\{\sum_{i,j}{\left(CC^{T}\right)}_{i,j}{\left(\mathrm{sgn}\left(\hat{\varphi }\right)\mathrm{sgn}\left({\hat{\varphi }}^{T}\right)\right)}_{i,j}\right\}$$

Note that:

$$\begin{array}{ccc} CC^{T} & = & \sum_{m=1}^{T}\sum_{l=1}^{T}{\hat{\phi }}^{\left(m\right)}{\left({\hat{\phi }}^{\left(m\right)}\right)}^{T}{\hat{\phi }}^{\left(l\right)}{\left({\hat{\phi }}^{\left(l\right)}\right)}^{T}\\ & = & \sum_{m=1}^{T}\sum_{l=1}^{T}\left(\sum_{n=1}^{N}{\hat{\phi }}_{n}^{\left(m\right)}{\hat{\phi }}_{n}^{\left(l\right)}\right){\hat{\phi }}^{\left(m\right)}{\left({\hat{\phi }}^{\left(l\right)}\right)}^{T} \end{array}$$

Then,

(A7) $$E\left\{{\left(CC^{T}\right)}_{i,j}{\left(\mathrm{sgn}\left(\hat{\varphi }\right)\mathrm{sgn}\left({\hat{\varphi }}^{T}\right)\right)}_{i,j}\right\}=\sum_{m=1}^{T}\sum_{l=1}^{T}\sum_{n=1}^{N}E\left\{{\hat{\phi }}_{n}^{\left(m\right)}{\hat{\phi }}_{n}^{\left(l\right)}{\hat{\phi }}_{i}^{\left(m\right)}{\hat{\phi }}_{j}^{\left(l\right)}\mathrm{sgn}\left({\hat{\varphi }}_{i}\right)\mathrm{sgn}\left({\hat{\varphi }}_{j}\right)\right\}$$

We analyze first the off-diagonal terms, i.e., i≠j, when $\left\lfloor \frac{i}{F}\right\rfloor \neq \left\lfloor \frac{j}{F}\right\rfloor$, and consider separately the cases where m≠l and m=l. For the first case, the right-hand side of (A7) can be written as:

$$\begin{array}{ccc} & & \sum_{m=1}^{T}\sum_{\overset{l=1}{l\neq m}}^{T}\sum_{\overset{n=1}{\left\lfloor \frac{n}{F}\right\rfloor \neq \left\lfloor \frac{i}{F}\right\rfloor \neq \left\lfloor \frac{j}{F}\right\rfloor }}^{N}E\left\{{\hat{\phi }}_{n}^{\left(m\right)}\right\}E\left\{{\hat{\phi }}_{n}^{\left(l\right)}\right\}E\left\{{\hat{\phi }}_{i}^{\left(m\right)}\mathrm{sgn}\left({\hat{\varphi }}_{i}\right)\right\}E\left\{{\hat{\phi }}_{j}^{\left(l\right)}\mathrm{sgn}\left({\hat{\varphi }}_{j}\right)\right\}\\ & + & F\sum_{m=1}^{T}\sum_{\overset{l=1}{l\neq m}}^{T}E\left\{{\left({\hat{\phi }}_{i}^{\left(m\right)}\right)}^{2}{\hat{\phi }}_{i}^{\left(l\right)}\mathrm{sgn}\left({\hat{\varphi }}_{i}\right)\right\}E\left\{{\hat{\phi }}_{j}^{\left(l\right)}\mathrm{sgn}\left({\hat{\varphi }}_{j}\right)\right\}\\ & + & F\sum_{m=1}^{T}\sum_{\overset{l=1}{l\neq m}}^{T}E\left\{{\left({\hat{\phi }}_{j}^{\left(l\right)}\right)}^{2}{\hat{\phi }}_{j}^{\left(m\right)}\mathrm{sgn}\left({\hat{\varphi }}_{j}\right)\right\}E\left\{{\hat{\phi }}_{i}^{\left(m\right)}\mathrm{sgn}\left({\hat{\varphi }}_{i}\right)\right\}\\ & = & 2F\sum_{m=1}^{T}\sum_{\overset{l=1}{l\neq m}}^{T}E\left\{{\left({\hat{\phi }}_{i}^{\left(m\right)}\right)}^{2}{\hat{\phi }}_{i}^{\left(l\right)}\mathrm{sgn}\left({\hat{\varphi }}_{i}\right)\right\}E\left\{{\hat{\phi }}_{i}^{\left(m\right)}\mathrm{sgn}\left({\hat{\varphi }}_{i}\right)\right\}\\ & = & \frac{2\mu_{Y}^{2}}{F}T\left(T-1\right) \end{array}$$

When $\left\lfloor \frac{i}{F}\right\rfloor \neq \left\lfloor \frac{j}{F}\right\rfloor$ and m=l, the right-hand side of (A7) can be written as:

$$\begin{array}{ccc} & & \sum_{m=1}^{T}\sum_{\overset{n=1}{\left\lfloor \frac{n}{F}\right\rfloor \neq \left\lfloor \frac{i}{F}\right\rfloor \neq \left\lfloor \frac{j}{F}\right\rfloor }}^{N}E\left\{{\left({\hat{\phi }}_{n}^{\left(m\right)}\right)}^{2}\right\}E\left\{{\hat{\phi }}_{i}^{\left(m\right)}\mathrm{sgn}\left({\hat{\varphi }}_{i}\right)\right\}E\left\{{\hat{\phi }}_{j}^{\left(m\right)}\mathrm{sgn}\left({\hat{\varphi }}_{j}\right)\right\}\\ & + & F\sum_{m=1}^{T}E\left\{{\left({\hat{\phi }}_{i}^{\left(m\right)}\right)}^{3}\mathrm{sgn}\left({\hat{\varphi }}_{i}\right)\right\}E\left\{{\hat{\phi }}_{j}^{\left(m\right)}\mathrm{sgn}\left({\hat{\varphi }}_{j}\right)\right\}+F\sum_{m=1}^{T}E\left\{{\left({\hat{\phi }}_{j}^{\left(m\right)}\right)}^{3}\mathrm{sgn}\left({\hat{\varphi }}_{j}\right)\right\}E\left\{{\hat{\phi }}_{i}^{\left(m\right)}\mathrm{sgn}\left({\hat{\varphi }}_{i}\right)\right\}\\ & = & \frac{1}{F^{2}}\left(T\left(N-2F\right)\mu_{Y}^{2}+2F\mu_{Y}^{2}\left(3T-1\right)\right) \end{array}$$

Now, we consider the rest of elements which satisfy that $\left\lfloor \frac{i}{F}\right\rfloor =\left\lfloor \frac{j}{F}\right\rfloor$, which are the *N* diagonal terms and the remaining N(F−1) off-diagonal ones. The right-hand side of (A7) is:

$$\begin{array}{ccc} & & \sum_{m=1}^{T}\sum_{l=1}^{T}\sum_{n=1}^{N}E\left\{{\hat{\phi }}_{n}^{\left(m\right)}{\hat{\phi }}_{n}^{\left(l\right)}{\hat{\phi }}_{i}^{\left(m\right)}{\hat{\phi }}_{i}^{\left(l\right)}\right\}=F\sum_{m=1}^{T}\sum_{\overset{l=1}{l\neq m}}^{T}E\left\{{\left({\hat{\phi }}_{i}^{\left(m\right)}\right)}^{2}\right\}E\left\{{\left({\hat{\phi }}_{i}^{\left(l\right)}\right)}^{2}\right\}\\ & + & \sum_{m=1}^{T}\sum_{\overset{n=1}{\left\lfloor \frac{n}{F}\right\rfloor \neq \left\lfloor \frac{i}{F}\right\rfloor }}^{N}E\left\{{\left({\hat{\phi }}_{n}^{\left(m\right)}\right)}^{2}\right\}E\left\{{\left({\hat{\phi }}_{i}^{\left(m\right)}\right)}^{2}\right\}+F\sum_{m=1}^{T}E\left\{{\left({\hat{\phi }}_{i}^{\left(m\right)}\right)}^{4}\right\}\\ & = & \frac{1}{F^{4}}\left(FT\left(T-1\right)+T\left(N-F\right)+3FT\right) \end{array}$$

where, for the last summand, we have used the fact that, for a zero-mean Gaussian random variable *X* with variance $\sigma^{2}$, $E\left\{X^{4}\right\}=3\sigma^{4}$. Then, the sum in (A7) is:

$$\begin{array}{ccc} & & \sum_{m=1}^{T}\sum_{l=1}^{T}\sum_{n=1}^{N}E\left\{{\hat{\phi }}_{n}^{\left(m\right)}{\hat{\phi }}_{n}^{\left(l\right)}{\hat{\phi }}_{i}^{\left(m\right)}{\hat{\phi }}_{j}^{\left(l\right)}\mathrm{sgn}\left({\hat{\varphi }}_{i}\right)\mathrm{sgn}\left({\hat{\varphi }}_{j}\right)\right\}\\ & = & N\left(N-F\right)\left[\frac{2\mu_{Y}^{2}}{F}T\left(T-1\right)+\frac{1}{F^{2}}\left(T\left(N-2F\right)\mu_{Y}^{2}+2F\mu_{Y}^{2}\left(3T-1\right)\right)\right]\\ & + & \frac{N}{F^{3}}\left(FT(T-1)+T(N-F)+3FT\right) \end{array}$$

and the resulting variance of $z_{j}$ is:

(A8) $$\begin{array}{ccc} \mathrm{Var}\{z_{j}\} & = & \left(N-F\right)\left(\frac{2\mu_{Y}^{2}}{F}T\left(T-1\right)+\frac{\mu_{Y}^{2}}{F^{2}}T\left(N-2F\right)+\frac{2\mu_{Y}^{2}}{F}\left(3T-1\right)\right)\\ & + & \frac{1}{F^{3}}\left(FT\left(T-1\right)+T\left(N-F\right)+3FT\right)-\frac{T}{F^{2}}\mu_{Y}^{2}{\left(N+\frac{F}{T}\left(T^{2}+T-1\right)]\right)}^{2}\\ & = & -\frac{\mu_{Y}^{2}}{FT}\left(FT^{4}+4FT^{3}+\left(N+F\right)T^{2}-4FT+F\right)+\frac{T}{F^{3}}\left(N+F(T+1)\right) \end{array}$$

#### Appendix A.4.2. Decomposition for Orthogonal Projectors

Now, for orthogonal projectors, we take a different route. Again, we define matrix $C≐[c_{0},\cdots ,c_{N}]$, and we note that $C={\hat{\Psi }}^{T}=\hat{\Psi }$. Recall also properties (13) and (14). We can collect all products $c_{j}^{T}\mathrm{sgn}\left(\hat{\varphi }\right)$, j=1,⋯,N in a vector obtained as $C^{T}\mathrm{sgn}\left(\hat{\varphi }\right)=\hat{\Psi }\mathrm{sgn}\left(\hat{\varphi }\right)$.

We are interested in computing the cross-product of this vector and $\hat{\varphi }$. Then, we can write:

$${\hat{\varphi }}^{T}\hat{\Psi }\mathrm{sgn}\left(\hat{\varphi }\right)=\frac{1}{F}{\hat{\varphi }}^{T}\mathrm{sgn}\left(\hat{\varphi }\right)$$

Assuming i.i.d. components in $\hat{\varphi }$, this implies that, for the orthogonal projectors, the cross-correlation of $c_{j}^{T}\mathrm{sgn}\left(\hat{\varphi }\right)$ and ${\hat{\varphi }}_{j}$ is the same as $\frac{1}{F}\mathrm{sgn}\left({\hat{\varphi }}_{j}\right)$ and ${\hat{\varphi }}_{j}$. We can then compute $E\left\{{\hat{\varphi }}_{j}\mathrm{sgn}\left({\hat{\varphi }}_{j}\right)\right\}=E\{|{\hat{\varphi }}_{j}\left|\right\}$. Modeling ${\hat{\varphi }}_{j}$ as N(0,T/(FN)), we find that $E\{|{\hat{\varphi }}_{j}\left|\right\}=\sqrt{\frac{2T}{\pi FN}}$. Furthermore, $E\{||\hat{\varphi }{\left|\right|}^{2}\}=E\left\{{\hat{\varphi }}^{T}\hat{\varphi }\right\}=E\left\{\varphi^{T}\Theta^{T}\Theta \varphi \right\}=\frac{T}{F}$.

The existence of a positive cross-correlation suggests writing $\frac{1}{F}\mathrm{sgn}\left({\hat{\varphi }}_{j}\right)=\alpha {\hat{\varphi }}_{j}+{\tilde{n}}_{j}$, with α a suitable positive constant and $\tilde{n}$ a zero-mean noise vector uncorrelated with $\hat{\varphi }$. Taking the cross-product and the expectation:

$$\frac{1}{F}E\left\{{\hat{\varphi }}^{T}\mathrm{sgn}\left(\hat{\varphi }\right)\right\}=\frac{N}{F}E\{|{\hat{\varphi }}_{j}\left|\right\}=\alpha E\{||\hat{\varphi }{\left|\right|}^{2}\}+E\left\{{\hat{\varphi }}^{T}\tilde{n}\right\}=\alpha E\{||\hat{\varphi }{\left|\right|}^{2}\}$$

Therefore, we find that:

(A9) $$\alpha =\frac{N\cdot E\{|{\hat{\varphi }}_{j}|\}}{F\cdot E\{||\hat{\varphi }|{|}^{2}\}}=\sqrt{\frac{2N}{\pi TF}}$$

Now, it is easy to measure the second order moment of ${\tilde{n}}_{j}$ since:

$$\begin{array}{ccc} E\{{\tilde{n}}_{j}^{2}\} & = & E\{{\left(\frac{1}{F}\mathrm{sgn}\left({\hat{\varphi }}_{j}\right)-\alpha {\hat{\varphi }}_{j}\right)}^{2}\}\\ & = & \frac{1}{F^{2}}+\alpha^{2}E\left\{{\hat{\varphi }}_{j}^{2}\right\}-\frac{2\alpha }{F}E\left\{{\hat{\varphi }}_{j}\mathrm{sgn}\left({\hat{\varphi }}_{j}\right)\right\}\\ & = & \frac{1}{F^{2}}\left(1-\frac{2}{\pi }\right) \end{array}$$

With the above characterization, we can see that:

$$\begin{array}{ccc} C^{T}\mathrm{sgn}\left(\hat{\varphi }\right) & = & F\alpha \hat{\Psi }\hat{\varphi }+F\hat{\Psi }\tilde{n}\\ & = & \alpha \hat{\varphi }+z \end{array}$$

Therefore, we have:

$$\begin{array}{c} \mathrm{Tr}\left[E\left\{zz^{T}\right\}\right]=F^{2}\cdot \mathrm{Tr}\left[E\left\{\hat{\Psi }\tilde{n}{\tilde{n}}^{T}\hat{\Psi }\right\}\right]=F\cdot E\left\{\mathrm{Tr}\left[\tilde{n}{\tilde{n}}^{T}\hat{\Psi }\right]\right\}=F\cdot E\left\{\sum_{i,j}{\left(\tilde{n}{\tilde{n}}^{T}\right)}_{i,j}{\left(\hat{\psi }\right)}_{i,j}\right\} \end{array}$$

Then,

$$E\left\{{\left(\tilde{n}{\tilde{n}}^{T}\right)}_{i,j}{\left(\hat{\psi }\right)}_{i,j}\right\}=E\left\{{\tilde{n}}_{i}{\tilde{n}}_{j}\right\}E\left\{\sum_{m=1}^{T}{\hat{\phi }}_{i}^{\left(m\right)}{\hat{\phi }}_{j}^{\left(m\right)}\right\}$$

When $\left\lfloor \frac{i}{F}\right\rfloor \neq \left\lfloor \frac{j}{F}\right\rfloor$, the expectation above is zero. For the NF remaining terms, that is, when $\left\lfloor \frac{i}{F}\right\rfloor =\left\lfloor \frac{j}{F}\right\rfloor$, we have ${\tilde{n}}_{i}={\tilde{n}}_{j}$ and ${\hat{\phi }}_{i}^{\left(m\right)}={\hat{\phi }}_{j}^{\left(m\right)}$. Thus:

$$E\left\{{\tilde{n}}_{j}^{2}\right\}\sum_{m=1}^{T}E\left\{{\left({\hat{\phi }}_{j}^{\left(m\right)}\right)}^{2}\right\}=\frac{1}{F^{2}}\left(1-\frac{2}{\pi }\right)\frac{T}{NF}=\frac{T}{NF^{3}}\left(1-\frac{2}{\pi }\right)$$

Finally, we can compute the variance of $z_{j}$ as follows:

(A10) $$\begin{array}{c} \mathrm{Var}\left\{z_{j}\right\}=\frac{1}{N}\mathrm{Tr}\left[E\left\{zz^{T}\right\}\right]=\left(1-\frac{2}{\pi }\right)\frac{T}{FN} \end{array}$$

### Appendix A.5. Adam: Analysis with Denoising and Watermarking

Here, we join the denoising and watermarking cost functions and follow a similar approach to that in Section 3.2.5. Let c be a random vector of length *N*, now we should build a random projection matrix, $\hat{\Phi }=\Theta \Phi$—with Gaussian or orthogonal projectors—, as a basis to generate samples of Ξ from (11) and to build c following (29).

We also consider that $\eta =\eta_{d}+\eta_{wm}$ is a random vector of length *N*, where $\eta_{d}$ and $\eta_{wm}$ represent the denoising and watermarking update terms, respectively. The components of $\eta_{d}$ can be computed from realizations of *D* and *V* like in Section 3.3 that is, $\eta_{d}=\delta /\left(\sqrt{\delta^{2}+\nu }\right)$. On the other hand, $\eta_{wm}$ has the same definition than in Section 3.2.5.

Let Ω be a random variable with the same distribution as $c^{T}\eta_{d}$ and let $M_{0}$, *P*, *Q* and *R* be random variables for which $m_{0,j}$, $p_{j}$, $q_{j}$ are $r_{j}$ are realizations, respectively. Then, we have:

$$\begin{array}{ccc} M_{0} & = & -\frac{\lambda }{2}\Xi -D\\ P & = & \Xi^{2}\\ Q & = & -\Xi \Omega -\Gamma \Xi (\alpha \Xi +Z)\\ R & = & \frac{1}{4}\left(\Omega^{2}+2\Gamma \left(\alpha \Xi +Z\right)\Omega +\Gamma^{2}{\left(\alpha \Xi +Z\right)}^{2}\right) \end{array}$$

Additionally, let *S* be a random variable for which $s_{j}$ is a realization. Now, it must include the contribution due to the denoising cost function so that:

$$\begin{array}{ccc} S & = & \frac{\lambda^{2}}{4}\left(P-\frac{\beta_{2}}{1-\beta_{2}}\mu Q+\frac{\beta_{2}+\beta_{2}^{2}}{{\left(1-\beta_{2}\right)}^{2}}\mu^{2}R\right)+D^{2}+V\\ & = & \frac{\lambda^{2}}{4}[\Xi^{2}+\frac{\beta_{2}}{1-\beta_{2}}\mu \left(\Xi \Omega +\Gamma \Xi (\alpha \Xi +Z)\right)\\ & + & \frac{\beta_{2}+\beta_{2}^{2}}{{\left(1-\beta_{2}\right)}^{2}}\frac{\mu^{2}}{4}\left(\Omega^{2}+2\Gamma \left(\alpha \Xi +Z\right)\Omega +\Gamma^{2}{\left(\alpha \Xi +Z\right)}^{2}\right)]+D^{2}+V \end{array}$$

Therefore, for a given realization (ξ, *z*, δ, ν) of (Ξ, *Z*, *D*, *V*), we must solve the following fourth degree equation to get samples of Γ (notice that, in order to generate samples, we must previously build the random vectors $\eta_{d}$ and c):

(A11) $$A_{1}\gamma^{4}+A_{2}\gamma^{3}+A_{3}\gamma^{2}+A_{4}\gamma +A_{5}=0$$

where:

$$\begin{array}{ccc} A_{1} & ≐ & \frac{\lambda^{2}}{4}\frac{\left(\beta_{2}+\beta_{2}^{2}\right)}{{\left(1-\beta_{2}\right)}^{2}}\frac{\mu^{2}}{4}{\left(\alpha \xi +z\right)}^{2} \end{array}$$

$A_{2}=A_{21}+A_{22}$ with $A_{21}$ and $A_{22}$ given by:

$$\begin{array}{ccc} A_{21} & ≐ & \frac{\lambda^{2}}{4}\frac{\beta_{2}}{\left(1-\beta_{2}\right)}\mu \xi \left(\alpha \xi +z\right)\\ A_{22} & ≐ & \frac{\lambda^{2}}{8}\frac{\beta_{2}+\beta_{2}^{2}}{{\left(1-\beta_{2}\right)}^{2}}\mu^{2}\left(\alpha \xi +z\right)\left(\Omega +\frac{\delta }{\sqrt{\delta^{2}+\nu }}\mathrm{sgn}\left(\xi \right)\left(\alpha \xi +z\right)\right) \end{array}$$

$A_{3}=A_{31}+A_{32}+A_{33}+A_{34}$ with the following definitions:

$$\begin{array}{ccc} A_{31} & ≐ & \frac{\lambda^{2}}{4}\xi^{2}\\ A_{32} & ≐ & \frac{\lambda^{2}}{4}\frac{\beta_{2}}{1-\beta_{2}}\mu \xi \left(\Omega +2\frac{\delta }{\sqrt{\delta^{2}+\nu }}\mathrm{sgn}\left(\xi \right)\left(\alpha \xi +z\right)\right)\\ A_{33} & ≐ & \frac{\lambda^{2}}{16}\frac{\beta_{2}+\beta_{2}^{2}}{{\left(1-\beta_{2}\right)}^{2}}\mu^{2}\left(\Omega^{2}+\frac{\delta^{2}}{\delta^{2}+\nu }{\left(\alpha \xi +z\right)}^{2}+4\frac{\delta }{\sqrt{\delta^{2}+\nu }}\mathrm{sgn}\left(\xi \right)\left(\alpha \xi +z\right)\Omega \right)\\ A_{34} & ≐ & \delta^{2}+\nu \end{array}$$

$A_{4}=A_{41}+A_{42}+A_{43}+A_{44}$ where:

$$\begin{array}{ccc} A_{41} & ≐ & \frac{\lambda^{2}}{2}\frac{\delta }{\sqrt{\delta^{2}+\nu }}\mathrm{sgn}\left(\xi \right)\xi^{2}\\ A_{42} & ≐ & \frac{\lambda^{2}}{4}\frac{\beta_{2}}{1-\beta_{2}}\mu \frac{\delta }{\sqrt{\delta^{2}+\nu }}\xi \left(2\mathrm{sgn}\left(\xi \right)\Omega +\frac{\delta }{\sqrt{\delta^{2}+\nu }}\left(\alpha \xi +z\right)\right)\\ A_{43} & ≐ & \frac{\lambda^{2}}{8}\frac{\beta_{2}+\beta_{2}^{2}}{{\left(1-\beta_{2}\right)}^{2}}\mu^{2}\frac{\delta }{\sqrt{\delta^{2}+\nu }}\Omega \left(\frac{\delta }{\sqrt{\delta^{2}+\nu }}\left(\alpha \xi +z\right)+\mathrm{sgn}\left(\xi \right)\Omega \right)\\ A_{44} & ≐ & 2\frac{\delta }{\sqrt{\delta^{2}+\nu }}\mathrm{sgn}\left(\xi \right)\left(\delta^{2}+\nu \right) \end{array}$$

Finally, $A_{5}=A_{51}+A_{52}+A_{53}+A_{54}$ with:

$$\begin{array}{ccc} A_{51} & ≐ & \frac{\lambda^{2}}{4}\xi^{2}\left(\frac{\delta^{2}}{\delta^{2}+\nu }-1\right)\\ A_{52} & ≐ & \frac{\lambda^{2}}{4}\frac{\beta_{2}}{1-\beta_{2}}\mu \frac{\delta^{2}}{\delta^{2}+\nu }\xi \Omega \\ A_{53} & ≐ & \frac{\lambda^{2}}{16}\frac{\beta_{2}+\beta_{2}^{2}}{{\left(1-\beta_{2}\right)}^{2}}\mu^{2}\frac{\delta^{2}}{\delta^{2}+\nu }\Omega^{2}\\ A_{54} & ≐ & \lambda \delta \xi \end{array}$$

Finally, we can generate samples of Δw as:

(A12) $$\Delta w\approx k\cdot \mu \cdot \left(\frac{\delta }{\sqrt{\delta^{2}+\nu }}+\gamma \cdot \mathrm{sgn}\left(\xi \right)\right)$$

## Appendix B. Verification of Assumptions

### Appendix B.1. Affine Growth Hypothesis for the Weights

In order to check the affine growth hypothesis that we introduced in (18), we extract the weight values from the embedding layer on each iteration. Figure A2 and Figure A3 show the evolution with *k* of four randomly selected weights when we respectively use: (i) SGD with orthogonal projectors, and (ii) Adam with Gaussian projectors. As is evident, the hypothesis holds quite accurately for these particular examples.

For a more general approach encompassing all weights, we carry out lineal regression on the evolution of each individual weight with *k*. Then, for each j∈{1,⋯,N}, we measure the correlation coefficient, $\rho_{j}$, between the observed values—the experimental weight values—and the predictor values. Figure A4 represents the Empirical Cumulative Distribution Function (ECDF) of $\rho_{j}$ when using SGD with orthogonal projectors and Adam with both Gaussian and orthogonal projectors. We can state that, when we use Gaussian projectors with Adam optimization, for 90% of the weights at the embedding layer $\rho_{j}>0.9410$ and for 80% of them $\rho_{j}>0.9970$. Moreover, the affine hypothesis is even stronger when using orthogonal projectors, both with SGD or Adam optimization, since for 95% of the weights at the embedding layer $\rho_{j}>0.9975$.

Because these percentages show a high-linear behavior, we can confirm the validity of the affine hypothesis for the weights that is key to our theoretical analysis.

### Appendix B.2. Negligibility of Weights at k = *0*

We first check the validity of the approximation $w^{\left(0\right)}\approx 0$ introduced in the analysis for SGD optimization. From (23), we can get η from the parameter settings detailed in Section 6.1 for the orthogonal case. We compute the gradient $g_{\nabla }≐\nabla_{w}f\left(w\right)$ following (20) and also its approximation $g_{\nabla }^{\prime }≐-\left(\lambda /2\right)\hat{\varphi }+\left(\lambda k\mu /4\right)\hat{\Psi }\eta$. In order to prove that $w^{\left(0\right)}\approx 0$, we can show that the preserved term, that is, the approximation $g^{\prime }$, is much larger than the discarded term $g_{\nabla }-g_{\nabla }^{\prime }=\left(\lambda /4\right)\hat{\Psi }w^{\left(0\right)}$, using the Normalized Mean Squared Error (NMSE). Let x be a *N*-length vector and $x^{\prime }$ its approximation, then the corresponding NMSE is defined as $\mathrm{NMSE}=||x-x^{\prime }{\left|\right|}^{2}/\left|\right|x^{\prime }{\left|\right|}^{2}$. Using this definition on $g_{\nabla }$ and $g_{\nabla }^{\prime }$, we get an NMSE of $3.6464\cdot 10^{-6}$, a very small value that supports our assumption $w^{\left(0\right)}\approx 0$.

Now, we check the same approximation for the Adam analysis. From (43), we can generate samples of γ using the parameter settings in Section 6.1. Let us recall the following definitions:

$$\begin{array}{ccc} m_{0} & = & -\hat{\varphi }+\frac{1}{2}\hat{\Psi }w^{\left(0\right)}\\ p_{j} & = & a_{j}-b_{j}^{T}w^{\left(0\right)}+\frac{1}{4}c_{j}^{T}w^{\left(0\right)}{\left(w^{\left(0\right)}\right)}^{T}c_{j}\\ q_{j} & = & -b_{j}^{T}\eta +\frac{1}{2}c_{j}^{T}w^{\left(0\right)}\eta^{T}c_{j} \end{array}$$

for j=1,⋯,N. Additionally, we define their corresponding approximations as:

$$\begin{array}{ccc} m_{0}^{\prime } & = & -\hat{\varphi }\\ p_{j}^{\prime } & = & a_{j}\\ q_{j}^{\prime } & = & -b_{j}^{T}\eta \end{array}$$

For Gaussian projectors, we get a NMSE of $3.9803\cdot 10^{-3}$, $5.2153\cdot 10^{-3}$ and $1.7148\cdot 10^{-3}$, for the approximations made for $m_{0}$, p and q, respectively. For orthogonal projectors, we get a NMSE of $3.6093\cdot 10^{-6}$, $5.1049\cdot 10^{-6}$ and $1.8010\cdot 10^{-6}$, for the approximations made for vectors $m_{0}$, p and q, respectively. As we see, these are small enough values to consider that our hypothesis $w^{\left(0\right)}\approx 0$ is reasonable for the analysis in Section 3.2.

## References

[1] He K., Zhang X., Ren S., Sun J. (2015). Delving Deep into Rectifiers: Surpassing Human-Level Performance on ImageNet Classification. Proceedings of the 2015 IEEE International Conference on Computer Vision (ICCV ’15).

[2] Nassif A.B., Shahin I., Attili I., Azzeh M., Shaalan K. (2019). Speech Recognition Using Deep Neural Networks: A Systematic Review. IEEE Access.

[3] Le Merrer E., Pérez P., Trédan G. (2017). Adversarial Frontier Stitching for Remote Neural Network Watermarking. arXiv.

[4] Adi Y., Baum C., Cissé M., Pinkas B., Keshet J. (2018). Turning Your Weakness Into a Strength: Watermarking Deep Neural Networks by Backdooring. arXiv.

[5] Uchida Y., Nagai Y., Sakazawa S., Satoh S. (2017). Embedding Watermarks into Deep Neural Networks. Proceedings of the 2017 ACM on International Conference on Multimedia Retrieval (ICMR ’17).

[6] Nagai Y., Uchida Y., Sakazawa S., Satoh S. (2018). Digital Watermarking for Deep Neural Networks. Int. J. Multimed. Inf. Retr..

[7] Cox I.J., Kilian J., Leighton F.T., Shamoon T. (1997). Secure Spread Spectrum Watermarking for Multimedia. IEEE Trans. Image Process..

[8] Bottou L., Saad D. (1998). Online Algorithms and Stochastic Approximations. Online Learning and Neural Networks.

[9] Kingma D.P., Ba J. Adam: A Method for Stochastic Optimization. Proceedings of the 3rd International Conference on Learning Representations (ICLR ’15).

[10] Rouhani B.D., Chen H., Koushanfar F. (2018). DeepSigns: A Generic Watermarking Framework for IP Protection of Deep Learning Models. arXiv.

[11] Balles L., Hennig P. Dissecting Adam: The Sign, Magnitude and Variance of Stochastic Gradients. Proceedings of the 2018 International Conference on Machine Learning (ICML ’18).

[12] Wilson A.C., Roelofs R., Stern M., Srebro N., Recht B. The Marginal Value of Adaptive Gradient Methods in Machine Learning. Proceedings of the 31st International Conference on Neural Information Processing Systems (NIPS ’17).

[13] Kullback S., Leibler R.A. (1951). On Information and Sufficiency. Ann. Math. Stat..

[14] Zhang K., Zuo W., Zhang L. (2018). FFDNet: Toward a Fast and Flexible Solution for CNN-Based Image Denoising. IEEE Trans. Image Process..

[15] Fan L., Zhang F., Fan H., Zhang C. (2019). Brief Review of Image Denoising Techniques. Vis. Comput. Ind. Biomed. Art.

[16] Ioffe S., Szegedy C. Batch Normalization: Accelerating Deep Network Training by Reducing Internal Covariate Shift. Proceedings of the 32nd International Conference on Machine Learning (ICML ’15).

[17] Sun S., Cao Z., Zhu H., Zhao J. (2020). A Survey of Optimization Methods From a Machine Learning Perspective. IEEE Trans. Cybern..

[18] Duchi J., Hazan E., Singer Y. (2011). Adaptive Subgradient Methods for Online Learning and Stochastic Optimization. J. Mach. Learn. Res..

[19] Tieleman T., Hinton G. (2012). Lecture 6.5—RMSProp, COURSERA: Neural Networks for Machine Learning.

[20] Wang T., Kerschbaum F. Attacks on Digital Watermarks for Deep Neural Networks. Proceedings of the 2019 IEEE International Conference on Acoustics, Speech and Signal Processing (ICASSP ’19).

[21] Burden R.L., Faires J.D. (2010). Numerical Analysis.

[22] Geyer C.J. (1992). Practical Markov Chain Monte Carlo. Stat. Sci..

[23] Cachin C., Aucsmith D. (1998). An Information-Theoretic Model for Steganography. Information Hiding.

[24] Comesaña P. Detection and information theoretic measures for quantifying the distinguishability between multimedia operator chains. Proceedings of the IEEE Workshop on Information Forensics and Security (WIFS12).

[25] Barni B., Tondi B. (2013). The Source Identification Game: An Information-Theoretic Perspective. IEEE Trans. Inf. Forensics Secur..

[26] Tassano M., Delon J., Veit T. (2019). An Analysis and Implementation of the FFDNet Image Denoising Method. Image Process. Line.

[27] Ma K., Duanmu Z., Wu Q., Wang Z., Yong H., Li H., Zhang L. (2017). Waterloo Exploration Database: New Challenges for Image Quality Assessment Models. IEEE Trans. Image Process..

[28] Franzen R. (1999). Kodak Lossless True Color Image Suite. http://r0k.us/graphics/kodak.

[29] Martin D., Fowlkes C., Tal D., Malik J. A Database of Human Segmented Natural Images and its Application to Evaluating Segmentation Algorithms and Measuring Ecological Statistics. Proceedings of the 8th Int’l Conf. Computer Vision (ICCV 2001).

[30] Wilson S.G. (1995). Digital Modulation and Coding.

